# Ultra-Deep Sequencing Reveals the Mutational Landscape of Classical Hodgkin Lymphoma

**DOI:** 10.1158/2767-9764.CRC-23-0140

**Published:** 2023-11-15

**Authors:** Felicia Gomez, Bryan Fisk, Joshua F. McMichael, Matthew Mosior, Jennifer A. Foltz, Zachary L. Skidmore, Eric J. Duncavage, Christopher A. Miller, Haley Abel, Yi-Shan Lee, David A. Russler-Germain, Kilannin Krysiak, Marcus P. Watkins, Cody A. Ramirez, Alina Schmidt, Fernanda Martins Rodrigues, Lee Trani, Ajay Khanna, Julia A. Wagner, Robert S. Fulton, Catrina C. Fronick, Michelle D. O'Laughlin, Timothy Schappe, Amanda F. Cashen, Neha Mehta-Shah, Brad S. Kahl, Jason Walker, Nancy L. Bartlett, Malachi Griffith, Todd A. Fehniger, Obi L. Griffith

**Affiliations:** 1 Department of Medicine, Division of Oncology, Washington University School of Medicine, St Louis, Missouri.; 2 McDonnell Genome Institute, Department of Medicine, Washington University School of Medicine, St Louis, Missouri.; 3 Siteman Cancer Center, Washington University School of Medicine, St Louis, Missouri.; 4 Department of Pathology and Immunology, Washington University School of Medicine, St Louis, Missouri.; 5 Department of Genetics, Washington University School of Medicine, St Louis, Missouri.

## Abstract

The malignant Hodgkin and Reed Sternberg (HRS) cells of classical Hodgkin lymphoma (cHL) are scarce in affected lymph nodes, creating a challenge to detect driver somatic mutations. As an alternative to cell purification techniques, we hypothesized that ultra-deep exome sequencing would allow genomic study of HRS cells, thereby streamlining analysis and avoiding technical pitfalls. To test this, 31 cHL tumor/normal pairs were exome sequenced to approximately 1,000× median depth of coverage. An orthogonal error-corrected sequencing approach verified >95% of the discovered mutations. We identified mutations in genes novel to cHL including: *CDH5* and *PCDH7,* novel stop gain mutations in *IL4R*, and a novel pattern of recurrent mutations in pathways regulating Hippo signaling. As a further application of our exome sequencing, we attempted to identify expressed somatic single-nucleotide variants (SNV) in single-nuclei RNA sequencing (snRNA-seq) data generated from a patient in our cohort. Our snRNA analysis identified a clear cluster of cells containing a somatic SNV identified in our deep exome data. This cluster has differentially expressed genes that are consistent with genes known to be dysregulated in HRS cells (e.g., *PIM1* and *PIM3*). The cluster also contains cells with an expanded B-cell clonotype further supporting a malignant phenotype. This study provides proof-of-principle that ultra-deep exome sequencing can be utilized to identify recurrent mutations in HRS cells and demonstrates the feasibility of snRNA-seq in the context of cHL. These studies provide the foundation for the further analysis of genomic variants in large cohorts of patients with cHL.

**Significance:**

Our data demonstrate the utility of ultra-deep exome sequencing in uncovering somatic variants in Hodgkin lymphoma, creating new opportunities to define the genes that are recurrently mutated in this disease. We also show for the first time the successful application of snRNA-seq in Hodgkin lymphoma and describe the expression profile of a putative cluster of HRS cells in a single patient.

## Introduction

Classical Hodgkin lymphoma (cHL) accounts for approximately 10% of newly diagnosed lymphoma cases. While most patients with cHL respond to front-line therapy and are cured, a subset relapses or are refractory, and remain a clinical challenge. Although brentuximab vedotin and immune checkpoint blockade have improved outcomes in relapsed/refractory cHL (1–3), improved prognostication and targeted treatment options continue to be an unmet need for this malignancy.

In the last decade, high-throughput genomic sequencing has provided insight into cancer pathogenesis and has facilitated the development of novel therapies. Unfortunately, the genomic characterization of cHL has been limited because of the paucity of the malignant Hodgkin and Reed Sternberg (HRS) cells, which generally comprise <5% of the tumor and are surrounded by an immunosuppressive non-neoplastic immune cell infiltrate (4). Only a few studies have investigated the genomic alterations representative of cHL. These studies have addressed the challenges of rare HRS cells in several ways, including the use of HRS-derived immortalized cell lines (5), HRS cell isolation from primary samples using laser capture microdissection or flow cytometric sorting (6–8), or through the study of circulating tumor DNA (9, 10). Patterns of recurrent somatic mutations have been identified within the NFκβ (e.g., *TNFAIP3*), JAK/STAT (e.g., *STAT6* and *SOCS1*), and the PI3K/AKT (e.g., *ITPKB*) signaling pathways (7, 9, 11–15). Despite this growing body of work, experimental limitations include the use of cell lines, small sample sets, and potential biases introduced by complex isolation techniques that require high HRS cell content. Because these techniques will not be feasible in a routine pathology laboratory, alternative approaches that utilize bulk lymph node tissue are required to perform large studies to correlate the impact of somatic mutations on clinical outcomes.

We performed ultra-deep exome sequencing to discover recurrent genomic events in 31 cHL bulk lymph node biopsies. An approach using multiple independent sequencing libraries per sample and custom variant filtering was developed to overcome the challenge of uncovering recurrent mutations among high-coverage, low variant allele frequency (VAF) sequencing data (16). Mutations were validated using an orthogonal error-corrected sequencing technique. This application of ultra-deep exome sequencing created a reliable and reproducible landscape of cHL somatic mutations, expanding our understanding of the genomic drivers and pathways important in cHL pathogenesis.

The size of Reed-Sternberg (RS) cells (up to 100 µm) makes this cell type a challenge for the microfluidic systems that accompany most single-cell sequencing technologies (17). In addition, HRS cell adherence to platelets and the T-cell rosetting that often surrounds HRS cells further challenge single-cell isolation techniques (18). To overcome the limitations of traditional single-cell isolation, we applied single-nuclei RNA sequencing (snRNA-seq) technology to a single patient in our cohort. Currently, an understanding of the patterns of gene expression in cHL remains underexplored. Gene expression profiling in microdissected primary cHL samples revealed changes in the regulation of pathways (e.g., JAK-STAT and NFκB) known to drive the pathogenesis of cHL (19). In addition, gene expression–based outcome predictor models have been proposed to predict overall survival (20, 21). Most recently, single-cell sequencing has been used to describe the tumor microenvironment of cHL and the importance of specific T-cell populations in cHL immune evasion (22, 23). However, a whole transcriptomic profile of HRS cells is lacking in the literature. Here, we have used a combination of newly discovered somatic variation and snRNA-seq to identify a putative cluster of HRS cells and have described patterns of differential gene expression. In addition, we have also assessed the remaining 29 samples with validated variants and have estimated that 12/29 samples (41%) have at least one variant with a high likelihood of detection in an snRNA experiment like the experiment we present here. We have also identified three additional samples (10%) from our cohort with a marginal likelihood of identifying an expressed variant. Our approach opens the door to the investigation of the full transcriptome of HRS cells. The snRNA-seq data lend support to the authenticity of the somatic variants described and provide a roadmap for studying the functional impact of the genomic changes we have observed.

## Materials and Methods

### Patient Sample Acquisition, Characteristics, and Ethical Considerations

All patients provided written informed consent for the use of their samples in sequencing as part of the Washington University School of Medicine (WUSM) Lymphoma Banking Program. The WUSM Institutional Review Board (IRB) approved protocols include: IRB 201108251, 201104048, 201110187. All human research activities are guided by the ethical principles in “The Belmont Report: Ethical Principles and Guidelines for the Protection of Human Subjects Research of the National Commission for the Protection of Human Subjects of Biomedical and Behavioral Research”.

We included all fresh-frozen excisional biopsies available in the bank from 2008 to 2015. Pathology review was performed on frozen lymph node samples to confirm the diagnosis of cHL (E.J. Duncavage and Y.S. Li). Both histochemistry and immunohistology were integrated to assess for CD15 and CD30 positivity and to quantify the number of RS cells in each sample. The approximate total of RS cells was determined using the number of RS cells per high-powered field (HPF) and total amount of tissue available per sample. Nodular lymphocyte-predominant Hodgkin lymphoma was not included. Nonmalignant samples were collected (skin punch biopsies) and were included for germline analysis. Frozen sections (tumor and skin) were cut and used for genomic DNA isolation. Most samples included in this study were untreated at the time of biopsy. In these cases, the sample utilized was the diagnostic excisional biopsy or core needle biopsy (27 untreated cases). There were also four relapses included. In two cases, the second relapse biopsy was sequenced and for the remaining two cases, the first and fourth relapse biopsy was sequenced. Basic demographics and clinical features are described in Table 1.

**TABLE 1:**
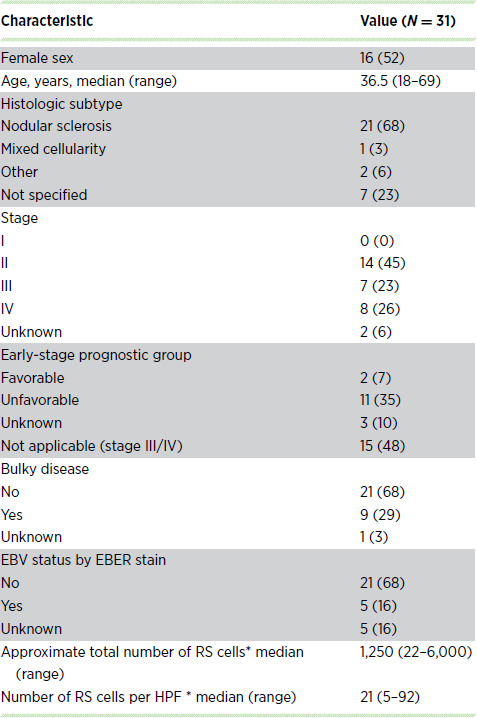
Patient characteristics and demographics

NOTE: Data are presented as No. (%) unless otherwise indicated. Histologic subtype *other* includes 1 patient where Hodgkin lymphoma and CLL were present (the CLL sample was excluded from all analyses) and a second patient that was designated as interfollicular. Histologic subtype *not specified* indicates that a cHL subtype was not provided. *Indicates five samples with no information regarding the number of RS cells.

### DNA Isolation, Library Preparation, and Sequencing

DNA was isolated using a Gentra Puragene kit at the Washington University Tissue Procurement Core facility. Automated dual indexed libraries were constructed with 30–250 ng of genomic DNA utilizing the KAPA HTP Library Kit (KAPA Biosystems) on the SciClone NGS instrument (Perkin Elmer) targeting 250 bp inserts. Three libraries were constructed per sample, each with a unique sample index. Libraries from the same patient (three from the normal and three from the tumor) were pooled prior to hybridization, yielding a 3 µg library pool. Each library pool was hybridized with the xGen Exome Research Panel v1.0 reagent (IDT Technologies) which spans a 39 Mb target region (19,396 genes) of the human genome. The concentration of each captured library pool was accurately determined through qPCR (Kapa Biosystems) to produce cluster counts appropriate for the HiSeq X platform (Illumina). A total of 2 × 151 bp paired end sequence data were generated with a target of approximately 100 Gb per sample and target mean coverage of approximately 1,000x.

### Single-nuclei Isolation and Library Prep (snRNA-seq)

One sample was piloted for snRNA-seq. Cryopreserved lymph node cells in single-cell suspension were thawed and prepared according to the 10x Genomics snRNA-seq protocol with the following modifications: Cells were lysed for 2–3 minutes in 1x lysis buffer with 10 mmol/L Tris-HCl (All Sigma: Trizma Hydrochloride, catalog no.: T2194), 10 mmol/L Sodium Chloride (catalog no.: 59222C), 3 mmol/L Magnesium Chloride (catalog no.: M1028), and 0.01% Non-Ident p40 (catalog no.: 74385) in Nuclease-free water, followed by centrifugation, and resuspension in nuclei wash and resuspension buffer [1% BSA (catalog no.: SRE0036)] in PBS with 0.2 U/µL of RNAase inhibitor (catalog no.: 3335399001). Cell lysis was validated using Acridine Orange (AO)/propidium iodide, with 72% of cells lysed. Nuclei were resuspended at 1,000 nuclei/µL in Nuclei Wash and Resuspension buffer and libraries were prepared using the 10x Genomics 52 snRNA-seq protocol (GTAC@MGI). Library preparation also included preparation for B-cell receptor (BCR) and T-cell receptor sequencing. The resulting 10x library was sequenced on an Illumina S4 flow cell (300 cycles targeting 100,000 reads/cell).

### HaloPlex Validation

Following variant calling, variant automated filtering, and manual review (see Supplementary Materials and Methods), we designed a custom capture reagent using the HaloPlex HS Target Enrichment System (Agilent Technologies) to validate the somatic variants. We included 1,842 out of 4,692 SNVs and INDELs that passed manual review. The variants that were included on the HaloPlex panel were all passing sites from all samples except where amplicons could not be designed (i.e., mitochondrial genes and repetitive regions), and a subset of sites from one sample (HL-513) with a very large number of variants. From this hypermutator, 834 out of 3,684 sites were included on the HaloPlex panel. We additionally tiled across 30 genes selected from the most recurrent genes in the cohort and genes known to be recurrently mutated in Hodgkin lymphoma (Supplementary Table S1; refs. 24, 25). The HaloPlex reagent was designed using the Agilent SureDesign platform (Supplementary Data S1). All probes were designed with two indices, a unique molecular barcode (UMI) to allow for error-corrected sequencing, and a sample index to allow for sample multiplexing (sample index).

HaloPlex libraries were created, sequenced, and processed using methods similar to previous reports, (26, 27) and the HaloPlex HS Target Enrichment System manufacturer protocol (Agilent Technologies). Up to 500 ng of genomic DNA was first digested using a mixture of restriction endonucleases in the HaloPlex kit. Library quality was assessed with the Agilent 2100 Bioanalyzer. Fragmented genomic DNA was then hybridized to the HaloPlex HS probe library. Hybridized genomic DNA fragments were ligated to close nicks in the probe-target DNA hybrids, captured with streptavidin, and amplified with PCR (22 cycles), creating read families, each with its own unique molecular barcode index. Library concentration was assessed with qPCR according to the manufacturer's protocol (Kapa Biosystems). Both tumor and normal samples were interrogated in the HaloPlex validation experiment.

Libraries were normalized, pooled, and sequenced on the HiSeq 4000. Eight samples were sequenced with two samples/lane and the remaining samples were sequenced with three samples/lane. HaloPlex sequence data were processed similar to the methods described previously (26). Barcoded FASTQ data were demultiplexed and then reads were trimmed using Flexbar (28). Trimming was performed to remove systematic errors introduced to the end of reads by HaloPlex chemistry. Reads were then aligned with Burrows-Wheeler Aligner (BWA) MEM v0.7.10-r789 (29).

All SNVs were evaluated using BarCrawler, a custom GATK-based tool. (https://github.com/abelhj/gatk/tree/master/public/external-example/src/main/java/org/abelhj). As described in Wong and colleagues (26), background noise calculation was performed on a position-by-position basis for each identified SNV. At each site, read counts were gathered from all other samples. A Fisher exact test, implemented in the R statistical environment, was used to compare the reference and variant read counts at a site with the number of reference and variant reads at that site in all other samples. The *P* value for this test was retained. Multiple testing correction was then applied with the *p*.adjust function [base R; default parameters—“holm” (30)]. Those variants with an adjusted *P* value of less than 0.1 were retained. The same process was then repeated with subsequent background calculations excluding all variants retained in previous rounds until no new variants were identified.

Following the final iteration of the background noise correction, SNVs with an adjusted *P* value of 0.05 were carried forward. Several other parameters were considered to validate a SNV. SNVs were required to have an error corrected depth e 100 reads in the tumor and the normal, and an adjusted *P* value >0.05 in the normal. In some instances, the normal sample did not have sufficient HaloPlex data to evaluate whether a site was present in the normal. In these cases, we designated the site as a “tumor-only” validated site if sufficient tumor depth (100x) was reached and a tumor *P* value d 0.05 was observed. Finally, as stated above only 834 of the 3,684 sites from the hypermutated patient were included on the HaloPlex reagent. We determined that only 12 of the 834 interrogated sites failed validation. Because this sample had a validation rate approximately 99% and few failing sites upon which a custom validation model could be trained, we simply considered the additional 2,850 SNVs from this patient as high-confidence sites and were included in all subsequent analyses.

Indels were assessed using “consensus bams”—alignments created on the basis of error corrected HaloPlex data. Consensus reads were generated from demuxed sequencing data via a wdl workflow that uses WalkerTRConsensus_wk5 (https://github.com/abelhj/gatk/blob/master/public/external-example/target/external-example-1.0-SNAPSHOT.jar).

Briefly, reads were trimmed of adapter sequences via cutadapt (-m 30 -u 3) and aligned with BWA-MEM. WalterTRConsensus_wk5 was then applied (-dcov 1000000 -maxNM5 -mmq 10) to generate consensus reads. Read families with less than 3 individual reads with the same UMI were removed and not used to create consensus reads. All INDELs were manually inspected using Integrative Genomics Viewer (IGV; ref. 31) and bam-readcount (32) was used to assess the number of variant supporting reads and the VAF for the variant. For INDELs, we required: a consensus bam tumor and normal depth > 5 consensus reads; > 1 consensus tumor read of support; < 20 consensus normal bam reads of support; consensus tumor VAF > 0.01%; a consensus normal VAF < 5%. Similar to the SNVs, if the normal consensus bam could not be evaluated, we required sufficient tumor bam depth (5 consensus reads), 1 variant read of support in the tumor and a tumor VAF > 0.01%.

### *De Novo* Variant Calling

Using the consensus bams described above, we attempted to call variants within the entire HaloPlex analysis space. To accomplish *de novo* variant calling, we created a custom low VAF variant calling pipeline by modifying an existing genome modeling system (16) variant calling workflow. Briefly, all postprocessing filters were removed, leaving only the filtering that occurs at the variant caller level using the default settings (https://github.com/fgomez02/analysis-workflows/blob/9c9e6a6a48eb321804ce772a2c2c12b4f2f32529/definitions/pipelines/detect_variants.cwl).

After variants were called, all *de novo* variants were filtered for basic variant quality. Variants with less than 5 consensus reads in the tumor and normal were removed. Sites were required to have a normal VAF < 5%, and a tumor VAF > 0.5%. We also required < 5 variant supporting reads (consensus reads) in the normal and e 2 variant supporting reads in the tumor. All *de novo* variants were annotated using the Ensembl Variant Effect Predictor (VEP) tool (Ensembl v93). Then, we filtered these variants by consequence using similar consequence filters described for the exome filtering pipeline. All variants that were previously discovered in the exome data were removed. We also intersected all remaining variants with the HaloPlex analysis space using bedtools (33), keeping only variants in regions where probes were designed. Following automated filters, all remaining variants were manually reviewed in IGV (31, 34). During manual review, the consensus tumor and normal bam files were loaded, as well as the exome tumor and bam file. Variants were passed if support was seen in the tumor consensus bam as well as the tumor exome bam file (and a lack of support was observed in the consensus and exome bam). Because we called and annotated new variants in the *de novo* variant calling exercise, we updated the consequence annotations of all exome variants using Ensembl VEP tool (Ensembl v93) for all sites that passed validation so that all sites would be annotated consistently. In addition, it should be noted that seven variants in *TCIRGI*, *CEP290*, *SMAD3*, *CEP131*, *COMP*, *LMTK3*, and *DOCK3*, that originally failed the orthogonal validation were called again in the *de novo* variant calling exercise. After manual review of all available data, these sites were rescued and included in the final analyses.

### Detection of Epstein-Barr Virus and Association of Epstein-Barr Virus Status with Mutation Burden

ISH for Epstein-Barr virus (EBV) mRNA (EBER) was used to detect the presence of EBV within each lymph node biopsy. Kallisto (35) was used to determine the presence of EBV DNA by performing competitive pseudoalignments to the human reference genome (GRCh38) and multiple viral genomes (EBV, human papillomavirus, and hepatitis B). To verify that our pseudoaligned reads were correctly aligned to EBV, we used BLASTn to search for all matching reads and verified that the EBV reference was among the highest concordances. Finally, to confirm the presence of EBV viral DNA, all samples were competitively aligned to the human and EBV reference genomes using BWA mem (29). These alignments were then visualized in IGV (31) where the presence of EBV reads was confirmed. A threshold of at least 2 reads with a MQ60 were required to confirm positivity.

We used a two-tailed *t* test to test whether the mutation burden (i.e., average number of mutations) across all samples that are EBV positive is significantly different from the mutation burden in EBV-negative cases. This test was performed using EBV status determined via EBER in situ hybridization and EBV status determined using the competitive alignment methods described above.

### Survival Analysis

For survival analysis, patients were stratified by mutation status (mutated vs. wild-type) for all genes mutated in 3 or more patients. Time-to-event analyses were performed using a log-rank test to identify significant progression-free survival (PFS) differences between patients with mutations in a particular gene and patients without mutations.

### Data Availability

The exome and snRNA seq data described in this article have been deposited in the NCBI sequence read archive (SRA) via dbGaP under the accession phs001229. The amplicons used for the orthogonal validation experiment are included as Supplementary Data.

## Results

### Patient Characteristics

Ultra-deep exome sequencing accompanied by orthogonal sequencing using HaloPlex technology was used to identify recurrent mutations in 31 fresh-frozen cHL biopsies with matched nonmalignant tissue. This cohort included samples from 27 (87%) patients with newly diagnosed and 4 (13%) patients with relapsed cHL with clinical characteristics shown in Table 1. Both histochemistry and immunohistology were integrated to assess for CD15 and CD30 positivity and to quantify the number of RS cells in each sample included here. The median number of RS cells per HPF was 21 (range: 5–92; Table 1; Supplementary Table S2).

### Genomic Data Generated and Coverage Statistics

**FIGURE 1:**
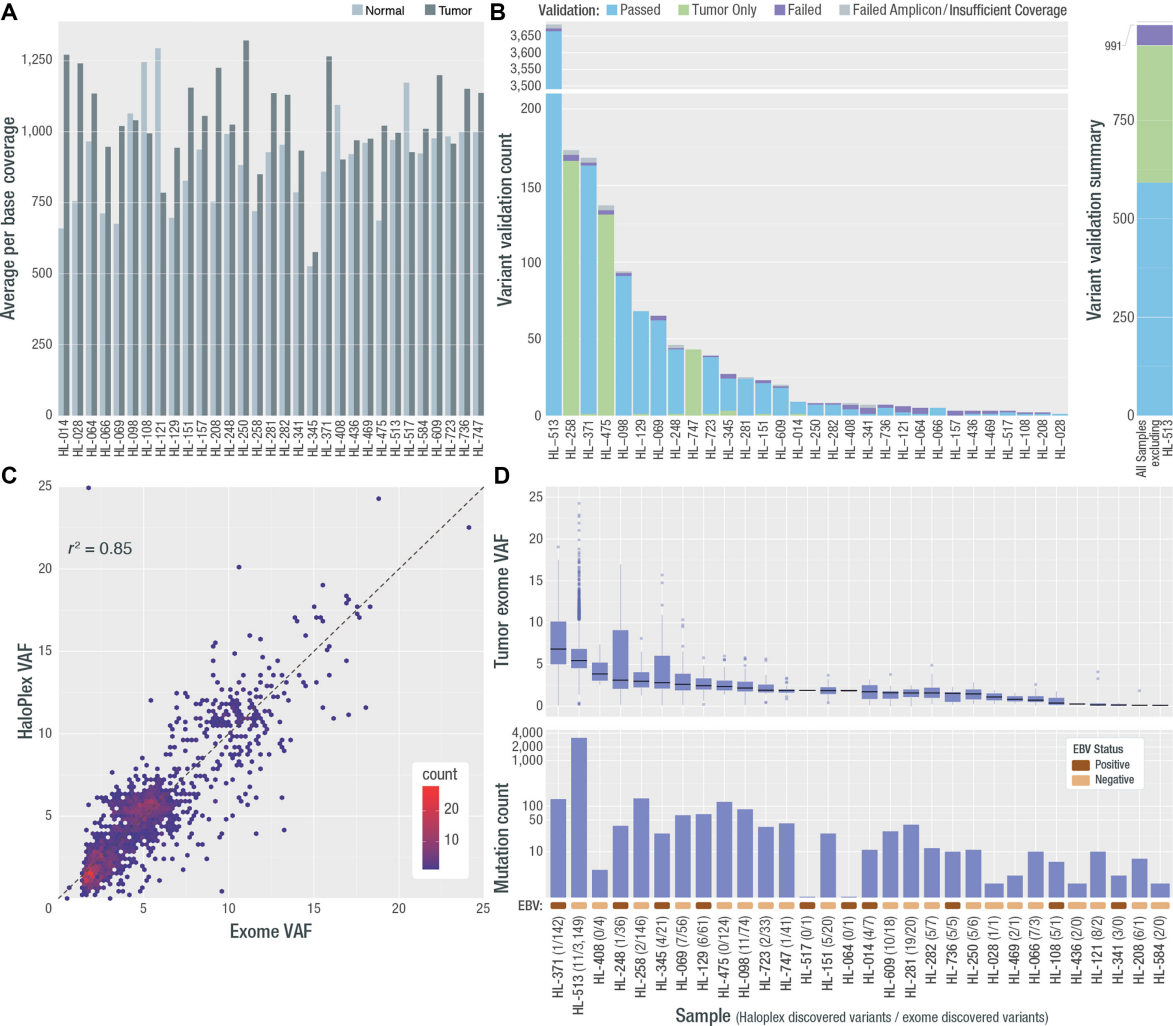
Deep exome coverage, validation rate, final variant count, and VAF per sample. **A,** Average per base coverage for targeted regions across each tumor and normal sample. **B,** A targeted orthogonal sequencing strategy (HaloPlex; Agilent) was used to validate all variants that passed filtering strategies for all samples (except for the hypermutated sample, for which a subset of variants were selected for validation—see Materials and Methods). The count of assayed variants that passed, failed, or were validated only in the HaloPlex tumor sample, is shown by sample as well as the number for which validation was not possible due to low tumor coverage or amplicon design failure. The overall count of variants that passed, failed, or received a “tumor only” validation status, excluding variants from HL-513 is shown at the right. Note: One patient (HL-584) did not have any variants pass filtering and review, therefore 30/31 patients were included in validation. **C,** Comparison of exome VAF and HaloPlex VAF is shown for samples with two or more variants. **D,** VAF and variant count for all variants across all samples used in all further analyses. Following each sample number is the number of HaloPlex discovered variants and exome discovered variants. In addition, a colored rectangle indicating the EBV stats (assessed using competitive alignment) is shown. Note: Variants from one patient failed validation (HL-157) and no further variants were called in *de novo* exercises. This patient was removed from all further analyses. In addition, the patient who did not have variants to validate, gained two variants in the *de novo* exercise and was included in the final cohort (HL-584).

We generated approximately 1,000x coverage exomes for both the fresh-frozen tumor and normal skin biopsy for all patients. The median depth of coverage per base, excluding duplicate reads, across all three libraries was 939x (range: 526–1,294x) for normal samples and 1,025x (range: 575–1,321x) for tumor samples (Fig. 1A; Supplementary Fig. S1). On average 111.11 Gb were generated per sample with an average on-target duplication rate of 16.5%, with 92% and 94% of bases covered at 200x, and 62% and 67% of the bases covered at 400x in the normal and tumor, respectively.

### Initial Variant Analysis and Variant Validation

We identified 4,692 SNVs and INDELs from the ultra-deep exomes that were considered for validation. One second relapse sample (HL-513) had 3,684 mutations, suggestive of a hypermutator phenotype (36, 37). Excluding this hypermutated patient, the median number of somatic mutations across the cohort was 11 and the mean was 32 (range: 0–148). The VAFs for all sites identified, were consistent with detection from rare HRS cells (mean VAF: 5.7%; median VAF: 5.2%; range: 0.5%–24.1%).

A HaloPlex panel, with molecular barcodes included for error correction, was designed to validate all variants from all patients, except a subset of sites from the hypermutated patients (see Materials and Methods). The final panel included 1,842 SNVs and INDELs. High depth sequencing data were generated with a median of 224,355,855 total reads/sample (range: 125,968,046–387,141,061 total reads) and a median error-corrected depth of coverage at 2,168x and 3,971x in the normals and tumors, respectively. The overall exome call validation rate by HaloPlex was 96.7% (1,754/1,814; Fig. 1B; Supplementary Fig. S2). This includes 1,405 fully validated sites and 349 “tumor only” validated (where the tumor variant was detected but matched normal data were unavailable). This rate excludes sites that could not be evaluated due to low tumor read depth (1.5%; 27/1,842 sites) or HaloPlex amplicon failure (0.05%; 1/1,842). If the “tumor only” validated sites are excluded from the count of validated sites, the validation rate for this experiment drops to 95.9% (1,405/1,465). We do observe a correlation between the number of cells/HPF and the sample validation rate (exclusive of HL-513, Pearson correlation = 0.50; Supplementary Fig. S3). These correlations are challenging, as counting the number of HRS cells is technically variable and non-uniform. An additional 2,850 sites from the hypermutated patient were not assayed because of HaloPlex size limitations. These sites were included in subsequent analyses because 98.5% (811/823) of tested HL-513 mutations, with sufficient depth, were validated (excluding 11 sites with low tumor depth; see Materials and Methods). The correlation of the HaloPlex and exome VAFs was *R*^2^ = 0.85 (*P* < 0.01), demonstrating significant concordance of VAF between the two platforms (Fig. 1C). A total of 60 sites (3.3%; 60/1,814) failed validation. The median VAF of sites that failed validation was 2.37% (range: 0.5%–13.53%) and the median tumor depth for sites that failed validation was 693 reads, (range: 54–6,287) suggesting that false positive variants occurred across the VAF spectrum of variants that were discovered and did not exclusively suffer from low coverage (Supplementary Figs. S4, S5, and S6). We conducted a *t* test comparing the distribution of coverage across sites that passed and failed validation and did not observe a significant difference between the level of exome coverage at sites that passed and failed validation (Supplementary Fig. S6). As a further exploration of the characteristics of failing variants, we observed a Spearman correlation (*R*_s_ = 0.64) between the total number of validated variants and the median exome VAF. However, despite this relationship we were able to validate >90 variants with a VAF d 1% (Supplementary Fig. S7). To further interrogate the etiology of the failing variants we examined the genomic position of each variant in the UCSC genome browser using the repeat masker, mappability, and the GC percent tracks. There were two variants that occurred within a microsatellite, one variant occurred in a region of low complexity, and one variant occurred within a SINE element. We also noted that the average %GC content for all failing variants is 62% (range: 0%–100%), which is higher than the genomic %GC content for the build38 reference (40.89%) and the corresponding mRNA %GC (<48%; ref. 38). The mappability for failing genomic variants was high with an average score of 0.988 (range: 0.583–1).

After validating exome-discovered mutations, new variant discovery was also attempted across our entire HaloPlex target space (Supplementary Table S1) including at known cHL hotspots (see Materials and Methods; Supplementary Table S3). From this *de novo* variant calling, 135 new variants were identified across the HaloPlex target region including seven variants at known Hodgkin lymphoma hotspots. These sites were called in 26 samples (Fig. 1D). These sites were likely missed in the exome data due to a small number of variant supporting reads in the original exome data (mean exome *de novo* variant allele count = 14; median exome *de novo* variant allele count = 5) compared with the variants that were originally called in the exome data (mean variant allele count = 70 and median variant allele count = 50). The small numbers of variant supporting reads for *de novo* variants were observed despite sufficient exome coverage (median coverage across *de novo* sites = 1,333) at these sites. In many cases, these variants were clearly present in the exome data and failed to meet our stringent criteria for variant calling. Future workflows could possibly increase sensitivity by lowering these thresholds. Our focus was on identifying high confidence variant calls for discovery of Hodgkin lymphoma driver genes and pathways. After updating the annotations of all potential variants (exome-validated and *de novo*—see Materials and Methods) the final annotated dataset consists of 4,116 non-synonymous coding somatic mutations that were carried forward for all subsequent analyses (Fig. 1D; Supplementary Table S4). The final cohort includes 30 individuals, as one patient had three exome-discovered variants that failed validation (patient HL-157). The median number of variants in this final validated dataset was 11, and the mean was 32.9 (range: 1–148), excluding the hypermutated patient, who contributed 3,160 variants to the final dataset. The mean and median VAFs were 5.6 and 5.1, respectively (range: 0.03–24.10; Fig. 1D).

### Recurrent and Significantly Mutated Genes

**FIGURE 2:**
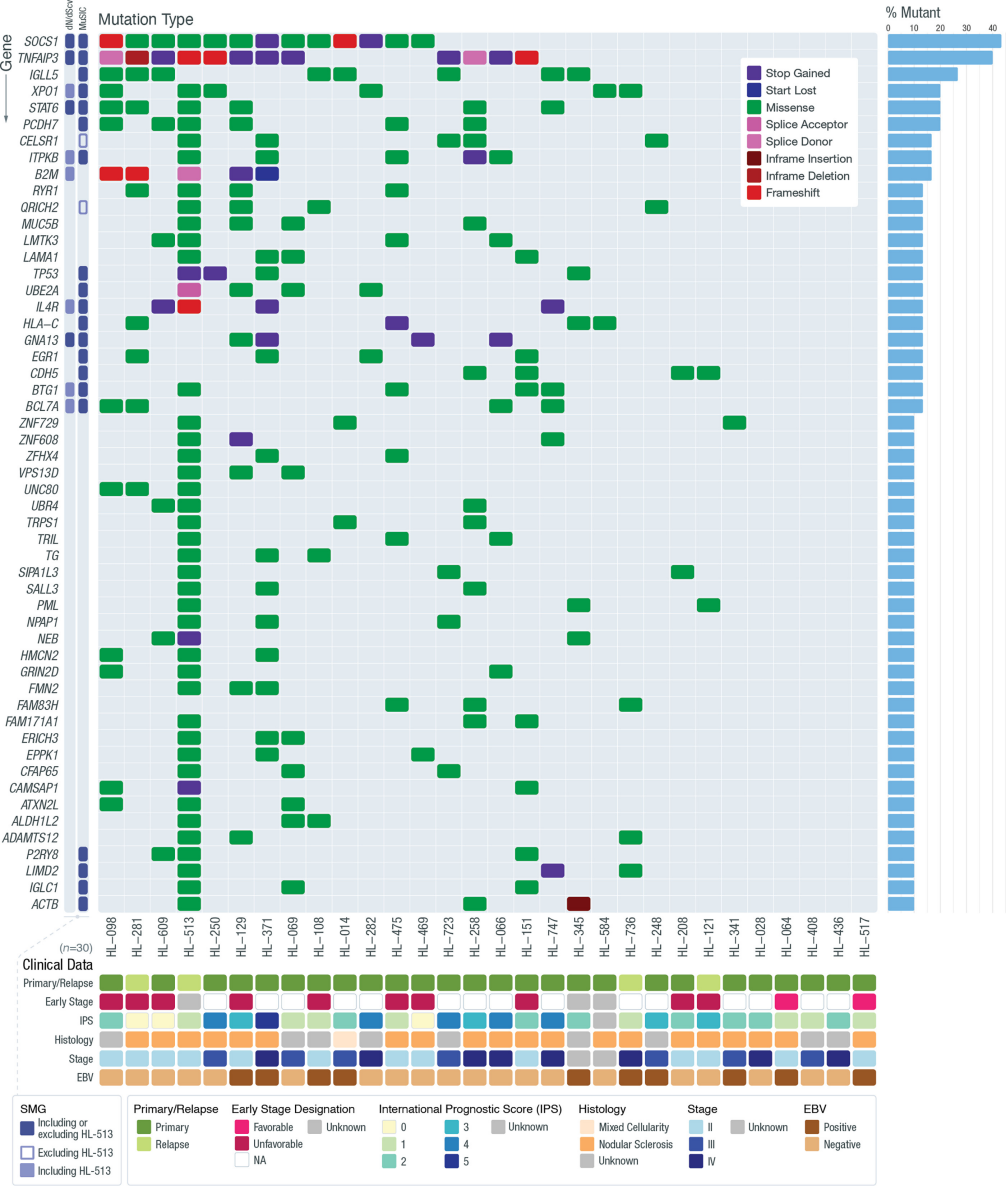
Recurrently mutated genes in Hodgkin lymphoma. The frequency and type of mutations affecting genes mutated in 3 or more patients in our cohort are shown in each row. Genes determined to be significantly mutated using MuSiC and/or dN/dScv (FDR < 0.05) are highlighted (SMG = significantly mutated gene). The open SMG shape indicates that a gene was determined to be an SMG only when HL-513 was excluded from the statistical analysis. The filled SMG shape indicates a gene that was determined to be an SMG only when HL-513 was included in the statistical analysis. The darkest SMG shape indicates that a gene was determined to be an SMG in all analyses (including and excluding HL-513). The bar graph on the right summarizes the frequency of mutations for that gene across the entire cohort. For genes with multiple mutations in a single patient, only one mutation type is shown, prioritized by the order listed in the legend.

A total of 3,168 somatically mutated genes were identified across all 30 samples, versus 732 mutated genes when the hypermutated patient was excluded. There were 263 genes with somatic mutations in at least 2/30 samples. The most recurrently mutated genes in our cohort are *SOCS1* (43.3%), *TNFAIP3* (40%), and *IGLL5* (26.7%) [Fig. 2; (percent recurrence)]. To identify significantly mutated genes (SMG), we took several approaches. As described in the Supplementary Materials and Methods section, we applied MuSiC (39) to the data with and without HL-513 and we also applied dN/dScv (40), again with and without HL-513 as a confirmatory strategy. When HL-513 is included in the MuSiC analysis, we identified 28 SMGs (Fig. 2; Supplementary Table S5). When HL-513 was excluded, a total of 32 genes were identified as potential SMGs (Fig. 2; Supplementary Table S5). Six genes (*AXDND1, OR13C2, RDH12, SCN9A SMAD3, STRAP)* were no longer significant and 10 additional genes (*MFHAS1, ZNF217, FBLN1, SBK1, HIST1H1B, SEL1L3, QRICH2, CELSR1, WDFY3, TRHR)* achieved an FDR <0.05. When we used dN/dScv to identify SMGs without HL-513 there were five genes with FDR < 0.05 (*TNFAIP3, SOCS1, GNA13, STAT6, CD83*; Fig. 2, Supplementary Table S5). When HL-513 was included in the dN/dScv analysis, six additional genes rose to significance (*BTG1*, *B2M*, *XPO1*, *IL4R*, *ITPKB*, and *BCL7A*; Fig. 2; Supplementary Table S5). All 11 SMGs identified by dN/dScv were identified in both iterations of MuSiC analysis. Because one goal of this study is discovery of novel genes involved in cHL pathogenesis, and the high mutation burden of HL-513 does not exclude those variants from contributing to informative genes characteristically mutated in Hodgkin lymphoma, we proceeded with the SMGs identified by MuSiC when all samples were included. In this analysis, the SMGs included several genes that have previously been shown to be mutated in cHL, including members of the JAK/STAT signaling pathway [e.g., *SOCS1* and *STAT6* (20%)] and the NFκB signaling pathway [e.g., *TNFAIP3* (40%) and *XPO1* (20%)]. Other SMGs known to be mutated in cHL include: *B2M* (16.7%), *ITPKB* (16.6%), and *GNA13* (20%). Components of the SWItch/Sucrose Non-Fermenting (SWI/SNF) complex including *BCL7A* (13%), *SMAD3* (6.6%), and *ARID1A* (6.6%), were also identified. *BCL7A* and *SMAD3* were identified as SMGs and are known to be involved in lymphomagenesis but have not been previously implicated in cHL. *ARID1A* was not among our SMGs but was mutated in three cases and has been identified by others to be recurrently mutated in cHL (7). A summary of genes found to be recurrently mutated in cHL across several recent studies of adult cHL (7–9, 24) is provided (Supplementary Fig. S8). To our knowledge, the following SMGs have not previously been reported in cHL: *AXDND1* (6.7%), *CDH5* (13.3%), *LIMD2* (10%), *OR13C2* (6.7%), *PCDH7* (20%), *RDH12* (6.7%), *SCN9A* (6.7%), and *STRAP* (6.7%).

Using the HL-513 inclusive SMG list determined by MuSiC, we examined patterns of co-occurrence and mutual exclusivity among the SMGs using the maftools R package (41). The results of this analysis identified 46 gene pairs with a co-occurrence Fisher *P* value <0.05 (Supplementary Table S6). Among these, eight pairs have an FDR <0.2 including: *AXDND1*/*STRAP*, *B2M*/*STAT6*, *B2M*/*TNFAIP3*, *ACTB*/*OR13C2*, *ACTB*/*RDH12*, *IGLC1*/*SCN9A*, *PCDH7*/*STAT6*, and *B2M*/*SOCS1*. In addition, B2M/STAT6 and B2M/TNFAIP3 have an FDR <0.1 (Supplementary Table S6; Supplementary Fig. S9). We did not identify significant evidence of mutual exclusivity, but this study is likely to be underpowered to see this sort of association. Association testing between mutation status and PFS, for our most recurrently mutated genes, did not yield significant results (Supplementary Table S7).

We did not observe a significant difference between mutation burden and EBV status determined by EBER (*P* = 0.11) or competitive alignment (*P* = 0.82; Supplementary Fig. S10). This contrasts with observations made by others (7, 8) who suggest a lower mutation burden is associated with EBV positivity. We believe this difference is likely due to low sample numbers, (i.e., if larger cohorts of patients with Hodgkin lymphoma were studied, we would have the potential to detect the association between EBV positivity and mutation burden). It should be noted that our methods for determining EBV status were mostly concordant; 5/5 EBER-positive samples appeared positive in the competitive alignments. Among the EBER-negative patients, 17/21 were also observed as negative using the alignment method. Four patients were determined negative by EBER but appeared positive using DNA alignments. There were no patients that appeared negative in the DNA alignments but were determined to be positive using EBER ISH. In addition, it should be noted that despite our threshold of EBV positivity (2 reads with MQ60; see Materials and Methods) most positive samples (7/10) had several hundred reads that aligned with the EBV reference sequence.

Previously unreported mutations in multiple cadherin genes were identified. There were 74 mutations in 42 different cadherin genes identified. The two cadherin SMGs were *PCDH7*, a protocadherin, and *CDH5*, a type II classical cadherin. Many of the cadherin mutations were only identified in the hypermutator patient; however, if these are excluded, 21 mutations were discovered across nine different cadherin-related genes. The percentage of the cohort that showed a mutation at these genes, exclusive of HL-513 is as follows: *CDH10* (3%), *CDH23* (3%), *CDH5* (16%), *CELSR1* (13%), *DSC2* (3%), *DSG3* (7%), *FAT3* (3%), *PCDH19* (3%), and *PCDH7* (16%). These mutations were found in 11 patients.

Four samples included here were biopsies taken when a relapse occurred. The samples included from patient HL-513 and patient HL-736 were taken at the second relapse. The sample taken from patient HL-281 was from the first relapse, and sample taken from patient HL-121 was at the fourth relapse. Among the relapse samples, excluding the hypermutated sample, we observed a median of 10 variants and a mean of 19 variants (range exclusive of hypermutated sample: 10–39). This is a slightly lower mutation burden than the non-relapse samples. However, the small number of relapse samples does not allow for an effective comparison of mutation burden between relapse and non-relapse patients. There were 27 genes with variants in at least one relapse sample (not including genes only mutated in the hypermutated sample; Supplementary Fig. S11). The genes included in Supplementary Fig. S11 are unique to the relapse samples. Although there are some genes that were mutated in 2 of the 4 relapse patients that have been shown to be associated with cell proliferation, especially PARP1 (42), the small number of relapse samples makes drawing any clear conclusions preliminary at best.

### Single-nuclei Transcriptome Profiling of a Hodgkin Lymphoma Biopsy

**FIGURE 3:**
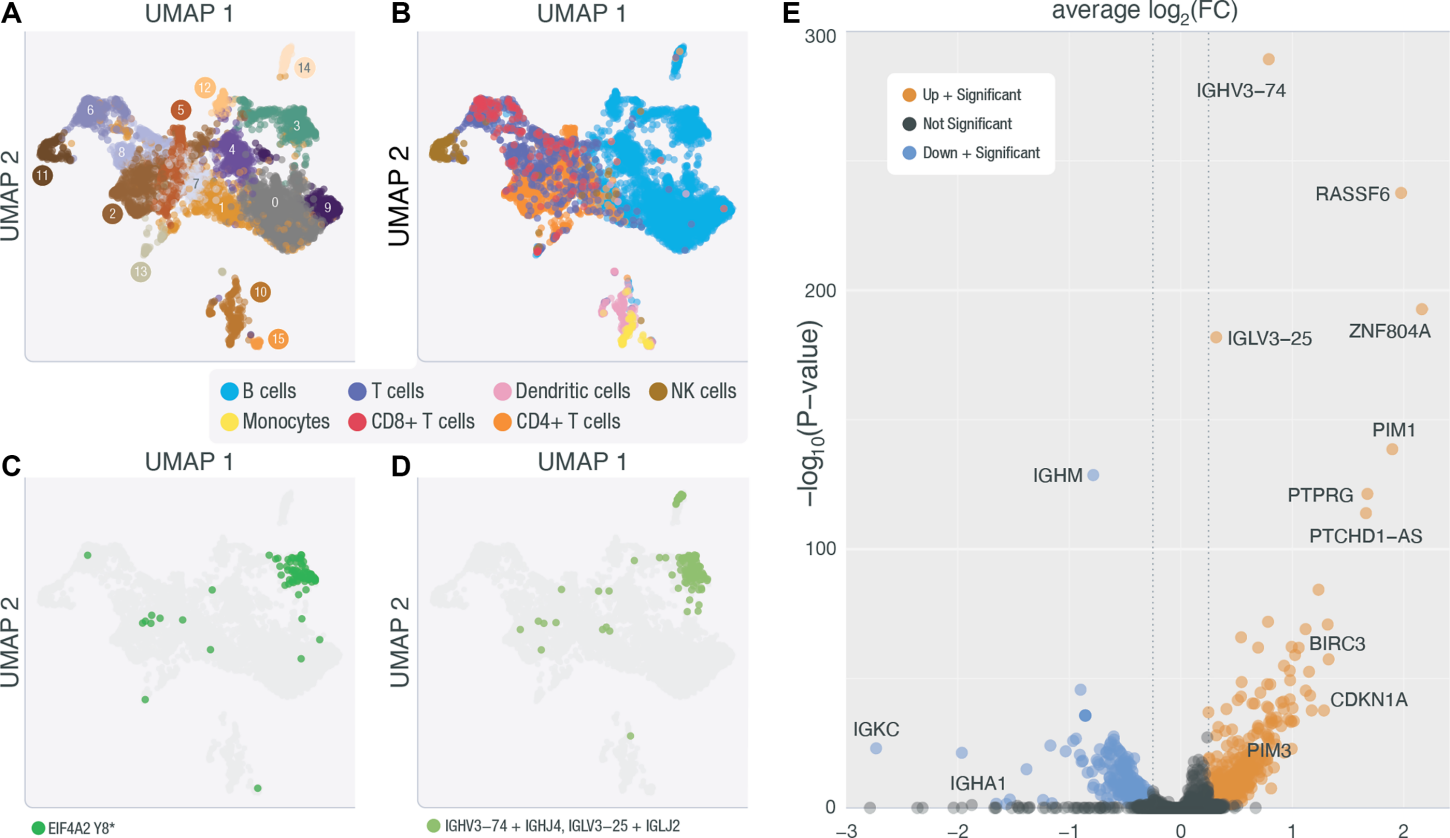
snRNA-seq of Patient HL-248. **A,** UMAP representation of unsupervised clustering of nuclei sequenced in HL-248. **B,** UMAP of HL-248 clusters with cell-type labels determined by celldex and SingleR. **C,** UMAP of HL-248 where highlighted nuclei are nuclei with a VAF > 0 for the *EIF4A2* variant uncovered in this sample's deep exome (chr3:186783634 T/A). **D,** UMAP of HL-248 where highlighted nuclei are nuclei that express an expanded BCR clonotype. **E,** Genes differentially expressed between cluster 3 and all other B cells are displayed as either downregulated with a significant *P* value (blue) or upregulated with a significant *P* value (orange).

As a proof-of-principle we attempted snRNA-seq on one Hodgkin lymphoma lymph node biopsy (HL-248), and we were able to identify 5,445 nuclei with a mean of 233,885 reads per nucleus. Using these data, principal component analysis was performed on the variable genes. These data were then used in Seurat's implementation of the Uniform Manifold Approximation and Projection (UMAP) algorithm for visualization. Unsupervised clustering was performed to identify nuclei with similar expression phenotypes (Fig. 3A). This clustering analysis identified 15 distinct clusters (Fig. 3A; Supplementary Table S8). Nuclei were labeled using SingleR (43, 44) and the Monaco (44) cell type reference provided in celldex and are indicated on the unsupervised UMAP. We identified the following cell types: B cells, T cells (CD4^+^ and CD8^+^), natural killer cells, dendritic cells, and monocytes (Fig. 3B; Supplementary Table S8). Using Vartrix (10x Genomics software) we searched for read support for the somatic SNVs identified in HL-248 deep exome data. We found support for one somatic SNV in *EIF4A2* (ENST00000323963.9:c.24T>A; Y8*). Most of the read support for this variant occurred in cluster 3, which was identified as a cluster among the B cells (Fig. 3C). Within cluster 3, 143 nuclei had a VAF >0 for the identified variant and the average VAF per cell was 77% (mean number of variant supporting reads = 2.5; range, 1–11; Fig. 3C). The identification of only a single expressed variant in this sample was not unexpected. Petti and colleagues (45) discuss the challenge of identifying expressed variants in RNA-seq from single cells extensively, with the most notable challenges being low transcript abundance (variable gene expression), relatively few reads per cell (sparse data), allelic dropout (failure to represent both alleles of a heterozygous site), and incomplete transcript coverage (extreme end bias), that is all inherent to the 10x single-cell approach. Using bam readcount (32), we assessed the 10x RNA sequence coverage of all 37 variant positions attributed to this sample. Only four variants had read coverage > 0; and the expressed variant we report here is the only site with a distance < 1 kb from the transcription start site, which is relevant here because the library we created is a 52 GEX library. For these reasons, we might only expect to find a small number of variants with relatively few reads of support in a subset of HRS cells.

To provide further evidence to support the identification of a malignant cluster of cells, we sequenced the BCR from all nuclei. BCR sequencing identified a dominant clonotype (IGHV3-74/IGHJ4: IGLV3-25/IGLJ2) that occurred in 6.2% of the nuclei identified as B cells with full-length productive CDR3 sequences (Fig. 3D; Supplementary Table S8). This clonotype was also found predominantly in cluster 3; we observed that 80.4% of the nuclei in cluster 3 have the dominant clonotype. We also see support for the expanded clonotype in cluster 14; this clonotype is supported in 24 (47%) nuclei in cluster 14. We used an expression correlation analysis (see Materials and Methods) to compare cluster 14 with the other B-cell clusters and found that cluster 14 has the highest expression correlation with cluster 3 (Pearson correlation = 0.76). However, we do not see evidence for the *EIF4A2* variant in cluster 14. The overall expression of *EIF4A2* in cluster 14 is lower than cluster 3 (Supplementary Fig. S12; average number of reads for *EIF4A2* across all cells with any expression of *EIF4A2* in cluster 3 = 211.9; cluster 14 = 48.7). Furthermore, among the limited number of *EIF4A2* reads within cluster 14, none cover the exon where the somatic variant was identified (Supplementary Fig. S13). This suggests that we do not have sufficient data to evaluate whether the identified somatic variant is also expressed in cluster 14. Further work is needed to understand the relationship between cluster 3 and cluster 14.

Next, we performed a differential expression analysis between cluster 3 and all other nuclei identified as B cells to identify genes that are differentially expressed in our presumed cluster of HRS cells (Fig. 3E). The differential expression analysis identified 613 genes that showed significant patterns of differential expression (*q* < 0.05). Among these, we identified genes known to be expressed or upregulated in cHL cell lines and primary samples including *PIM1* (*q* = 1.15 × 10^−131^), *PIM3* (*q* = 2.52 × 10^−47^), and *CD83* (*q* = 4.90 × 10^−21^). We also showed that *BCL2* is upregulated (*q* = 7.10 × 10^−16^; Supplementary Table S9; Fig. 3D), which is known to be upregulated in other B-cell lymphomas. These data serve as preliminary evidence to suggest that snRNA-seq can be used in the context of cHL biopsies and as an additional validating experiment to support that the variants we have identified using our ultra-deep methods can be attributed to HRS cells. If we assume that read support observed for this pilot case is representative of a typical snRNA cHL experiment, and interrogate the variable positions detected in the other 29 samples, we estimate that 12 of 29 patients with validated variants (41.4%) would have a high likelihood of at least one detectable variant, allowing for the identification of HRS cell clusters in additional patients (see Materials and Methods).

### Mutation Signature Analysis and Activation-induced Cytidine Deaminase (AID) Targets

**FIGURE 4:**
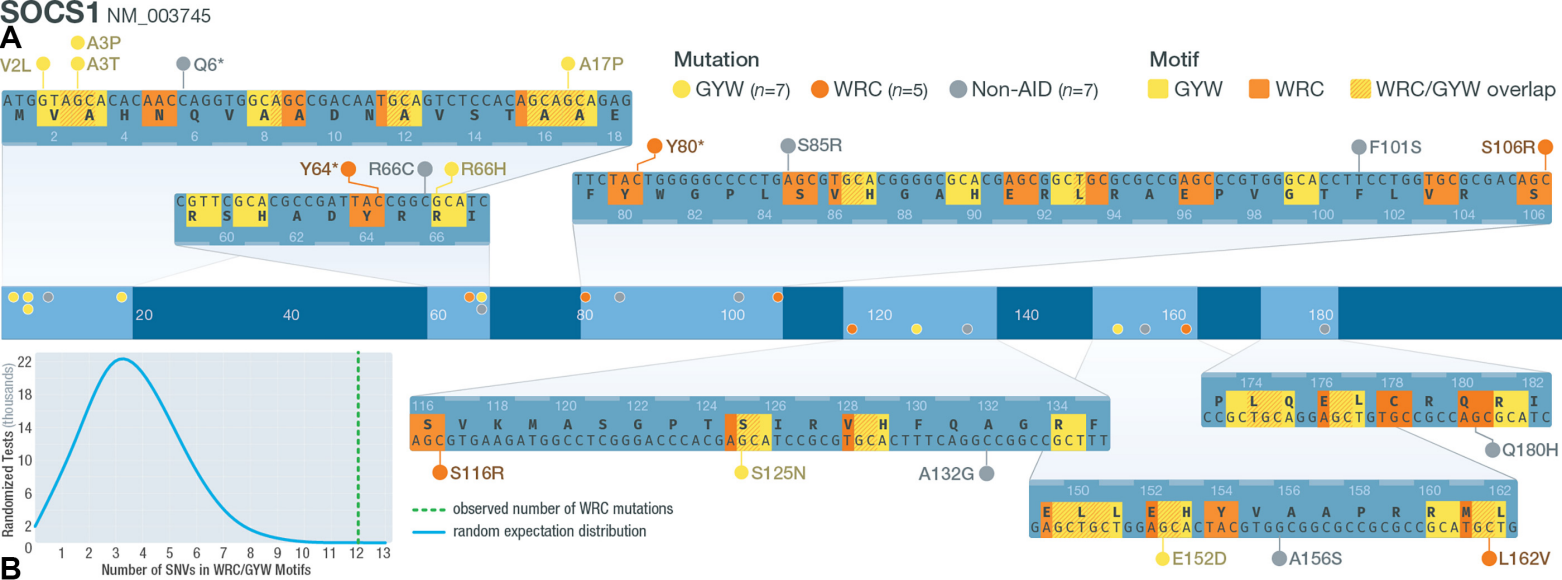
Mutations in AID motifs at SOCS1. **A,** Schematic of *SOCS1* gene structure. Middle track shows where *SOCS1* SNVs are located. Zoomed-in tracks (top and bottom) show the location of WRC/GYW motifs and the mutations that lie within or outside motifs (W = A/T; R = A/G; Y = C/T). **B,** Distribution of counts of simulated mutations observed at *SOCS1* that were in WRC motifs. The actual observed number of WRC SNVs (12/19) is shown using a green dashed line.

To further understand the etiology of somatic mutations in cHL, a mutation signature analysis was conducted in the patients with 50 or more SNVs (7 patients; see Supplementary Materials and Methods). The most prevalent COSMIC mutation signatures were: 39, 1, 6, 9, 30, 29, 51, 7a, 85, and signature 5 was observed in HL-371 (Supplementary Fig. S14). We observed the presence of COSMIC signature 9 and 85 in several patients (Supplementary Fig. S14). These signatures are associated with the activity of AID. In a recent analysis of the chronic lymphocytic leukemia (CLL) mutational landscape, two AID signatures were observed (46); one characterized by a canonical AID signature that includes C to T/G mutations at the WRC/GYW AID hotspot, as well as a noncanonical AID signature that includes A to C mutations at WA motifs, which is consistent with COSMIC signature 9. Although our analysis suggests that signature 9 and 85 (noncanonical AID) are common in our cohort, other studies (7, 47) have shown that canonical AID is characteristic of cHL. Indeed, Maura and colleagues (2019; ref. 48) suggest that canonical and noncanonical AID signatures are often observed together in other lymphoproliferative disorders. We acknowledge that within the undetermined mutation signatures (accounting for an average of 27% of the trinucleotides identified per sample; Supplementary Fig. S14), there could be evidence of canonical AID activity that our method failed to identify. Therefore, we hypothesized an additional method not limited by known mutation signatures or sample number could identify evidence of canonical AID activity. To address whether our data also include a canonical AID signature, a list of genes known to be significant targets of off-target canonical AID activity in diffuse large B-cell lymphoma (DLBCL) and follicular lymphoma (FL) was generated (49, 50). Our analysis (see Supplementary Materials and Methods) revealed 24 canonical AID-target genes were mutated in at least one non-hypermutated patient sample. Of these, eight genes had SNVs (23 SNVs total) located in the known WRC/GYW canonical AID target motif, including: *SOCS1*, *IGLL5, ARID5B, CD83, HIST1H2AL, ZFP36L1, HIST1H1B*, and *HIST1H1C*. Of the 19 SNVs identified in *SOCS1*, 12 were within WRC/GYW motifs (Fig. 4A). Four of nine SNVs in *IGLL5* and two of two SNVs in *HIST1H1B* were within a WRC/GYW motif. At *ARID5B, ZFP36L1, HIST1H2AL,* and *HIST1H1C*, one SNV was identified at each locus in a WRC/GYW motif (1/1, 1/3, 1/1, and 1/1 SNVs in each gene, respectively). When we tested whether the number of SNVs identified within WRC/GYW motifs was significantly different from random expectations by random simulations, we found on average, each *SOCS1* simulation had 3.66 mutations that met the criteria for a potential mutation generated by aberrant AID activity. Only 9/100,000 simulations had at least 12 variants, as we observed in our actual cohort (*P* = 0.0009; Fig. 4B). The simulated *IGLL5* dataset identified only 47/100,000 tests with at least four SNVs that could result from aberrant AID activity (*P* = 0.0005). We assessed the enrichment of off-target AID mutations at the other loci we identified, and three additional genes exhibited significant enrichment for AID mutations: *ARID5B* (*P* = 0.0083), *HIST1H1B* (*P* = 0.017), and *ZFP36L1* (*P* = 0.014). A table with the expected number of AID SNVs for *SOCS1*, *IGLL5*, *HIST1H1B*, *ARID5B,* and *ZFP36L1* is included (Supplementary Table S11). We also asked whether the overall number of mutations we saw across the 24 potential AID targets was different from random expectations. The results of this analysis showed that, we would expect to observe 6.36 total mutations meeting our AID target criteria. We did not observe any permutations with a total of 23 mutations (*P* < 0.00001), suggesting that the overall pattern of off-target AID activity is significantly different from random expectations. This suggests that canonical AID activity is indeed present at previously identified loci of AID.

### JAK/STAT Signaling Mutations

**FIGURE 5:**
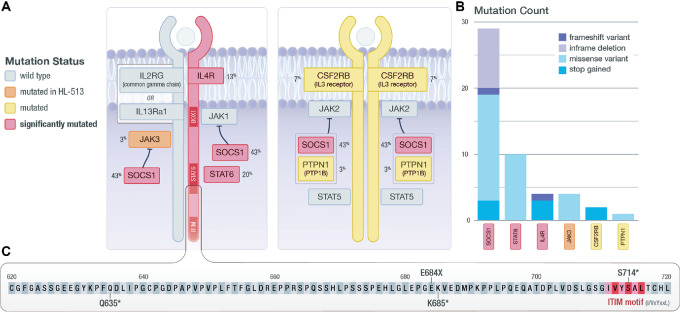
JAK/STAT signaling. **A,** Diagram of components of the JAK/STAT signaling cascade. Identified SMGs are shown in red; genes mutated in at least one non-hypermutated sample are shown in yellow; genes mutated only in the hypermutated sample (HL-513) are shown in orange. The gene mutation frequency across the cohort is shown as a percent. **B,** The total number and type of mutation observed are shown. **C,** Zoomed-in view of the C-terminal region of IL4R, where the observed truncating mutations are located. Also shown is the proximity of identified mutations to an ITIM motif that may impact STAT6 activation.

Mutations were identified in 22 genes from the JAK/STAT signaling pathway, with 50% of our cohort (15/30) having at least one somatic mutation in a JAK/STAT signaling gene (Fig. 5A and B). Several novel stop gain and frameshift mutations were discovered, clustered in the genomic region that encodes the cytoplasmic region of IL4R that contains an immunoreceptor tyrosine-based inhibitory motif (ITIM). These mutations were downstream of the box1 motif (JAK1 interaction region) and the amino acids that are thought to be required for STAT6 interaction (Fig. 5C; ref. 51). The potential loss of function mutations may represent an additional mechanism to promote cHL proliferation in response to IL4 stimulation via mutation of normally inhibitory ITIMs. Recently, these mutations were confirmed in a separate cohort of 119 patients with cHL profiled through cell-free DNA (52). Further work is necessary to understand the function of these mutations, but the additional data do support the possibility that these mutations have a functional role in this disease.

Two nonsense mutations were identified in the cytoplasmic regions of CSF2RB (IL3RB). This gene encodes the common β chain (CD131) that associates with the IL3, IL5, and the GMCSF alpha receptors (Fig. 5). CSF2RB has been shown to be recurrently mutated in Hodgkin lymphoma cell lines (5) and Hodgkin lymphoma primary samples (7, 53). The nonsense mutations we identified at this locus are beyond the JAK2 box 1 motif (amino acids 474–482). Because a truncated isoform of the common β chain may be related to the pathogenesis of acute myeloid leukemia (AML; ref. 54), and mutations in the cytoplasmic region have been associated with growth in T-cell acute lymphoblastic leukemia (55), it is possible that these mutations are related to cHL pathogenesis. Further work is necessary to address these hypotheses.

### Mutations in Genes Regulating Hippo Signaling

**FIGURE 6:**
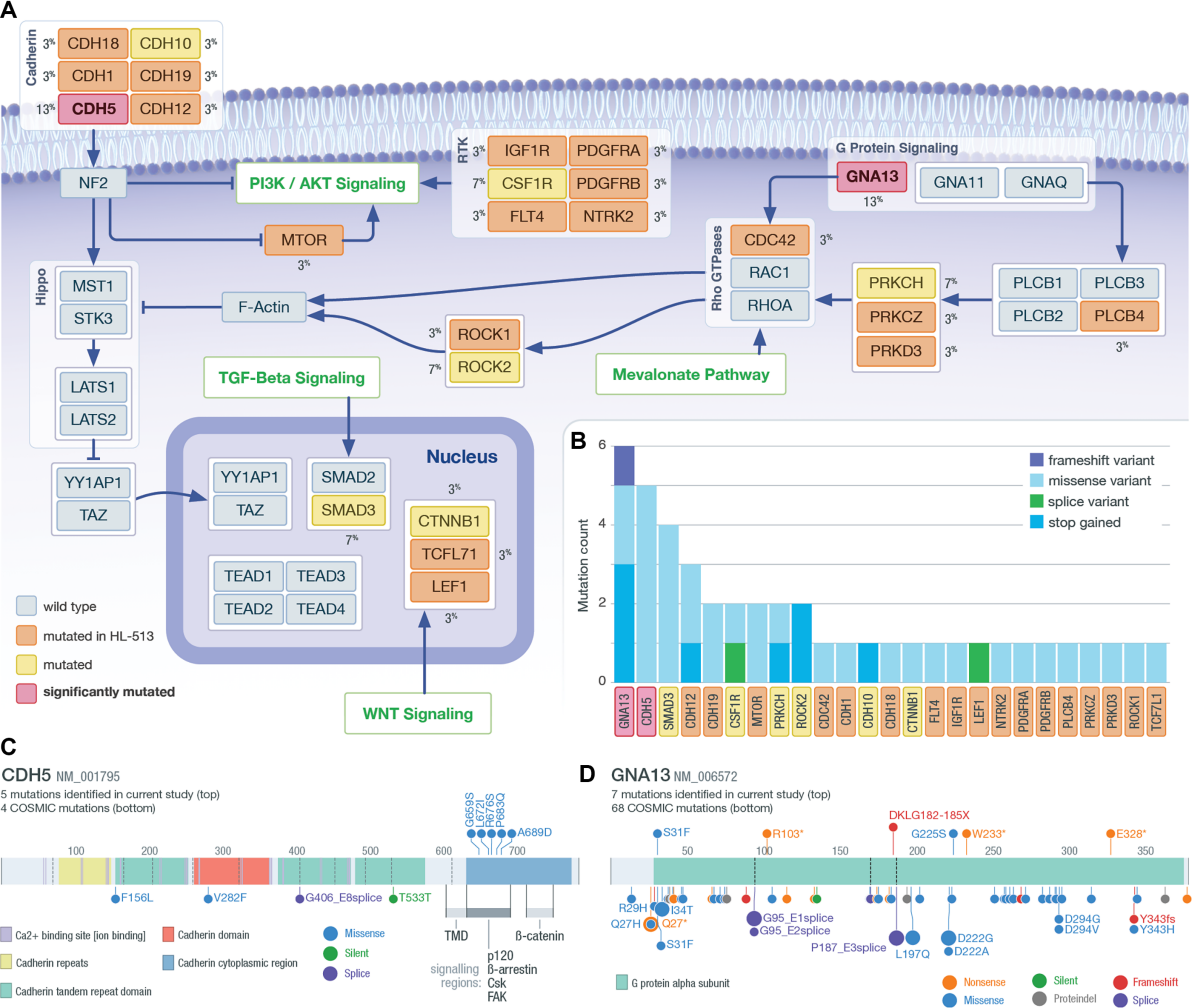
Regulating Hippo. **A,** Diagram of pathways that regulate Hippo signaling. Identified SMGs are shown in red; genes mutated in at least one non-hypermutated sample are shown in yellow; genes only mutated in the hypermutated sample (HL-513) are shown in orange. The gene mutation frequency across the cohort is shown as a percent. The pathways labeled in green indicate larger pathways not shown in this diagram. **B,** The total number and type of mutation observed are shown in the inset bar chart. **C,** Lolliplot of *CDH5*; mutations identified in the current study are shown on the top and COSMIC mutations found in lymphoid tissue on the bottom. **D,** Lolliplot of mutations identified at *GNA13*; mutations identified in the current study are shown on the top and COSMIC mutations found in lymphoid tissue on the bottom.

Somatic mutations in 31 genes involved in pathways regulating Hippo/TAZ/YAP or directly interacting with the Hippo cascade were identified (Fig. 6A and B). These genes are mutated in 40% (12/30) of our cohort. Because the regulation of the Hippo/TAZ/YAP pathway is a novel cHL-associated pathway, we compared the distribution of VAFs in this pathway with the distribution of VAFs associated with JAK/STAT variants. The variants in these pathways have similar VAF distributions (Supplementary Fig. S15). The JAK/STAT variants have a mean VAF of 4.4% and the Hippo/TAZ/YAP regulation variants have a mean VAF of 4.8%. These data suggest that the variants in the Hippo/TAZ/YAP regulator pathway have a similar level of support as the variants in well-established pathways like JAK/STAT. Two SMGs, *CDH5* and *GNA13*, are among this group. CDH5/VE-cadherin has not been previously described as a driver of cHL. VE-cadherin is linked through its cytoplasmic tail to adherens junction (AJ) proteins, p120, beta-catenin, and plakoglobin (56). The mutations identified here span amino acids 650–680, which could impact the p120 and beta-arrestin association regions of VE-cadherin that are vital for the stability of catenin-cadherin complexes (Fig. 6C). It has been shown that disruption of VE-cadherin clustering or suppression of VE-cadherin expression results in the nuclear localization of YAP and the promotion of cell proliferation (57, 58). Additional data are necessary to address whether the mutations identified here impact catenin–cadherin complexes.

G±13 (encoded by *GNA13*) is a G-protein coupled receptor known to be mutated in cHL (7–9, 24). G±13 is also involved in G-coupled signaling that activates Rho GTPases, which subsequently activates Rho-associated protein kinase I and II (ROCK1/2). This leads to actin cytoskeletal tension and has been shown to negatively regulate YAP/TAZ phosphorylation (59–62). We observed several missense, frameshift, and nonsense mutations, consistent with previous observations in cHL, (7–9) Burkitt's lymphoma, and DLBCL (63, 64). Frameshift and nonsense mutations may cause loss of function of G±13, which is unlikely to promote TAZ/YAP signaling (63, 65). However, two missense mutations were observed, including one located at G225S, which is similar to a dominant-negative mutation at G225A (64, 66) and close to Q226L, which is known to cause constitutive activation of G±13 (Fig. 6D; refs. 60, 67, 68). In addition to the likely loss-of-function consequences of the *GNA13* mutations observed, we identified other variants that may impact Hippo signaling either through GTPases or other pathways, including mutations at *PRKCH*, *ROCK2,* and *CSF1R* (69–71).

### Additional Pathways Targeted by Somatic Mutations

We identified mutations in 59 genes that are involved in MAPK signaling pathways; 43% (13/30) of the cohort had at least one mutation in a gene annotated to a MAPK signaling cascade (Supplementary Fig. S16). In addition, 30% (9/30) of our cohort had a mutation in a gene mapped to phosphatidylinositol signaling, including the SMG *ITPKB*. Like Tiacci and colleagues (8) *ITPKB* was mutated in 16.6% of our cohort (Supplementary Fig. S17). Several missense mutations and only one nonsense mutation (p.Y4*) were discovered, which contrasts with the high frequency of truncating mutations reported previously (8).

### Germline Mutation and Microsatellite Instability in Hypermutated Patient

**FIGURE 7:**
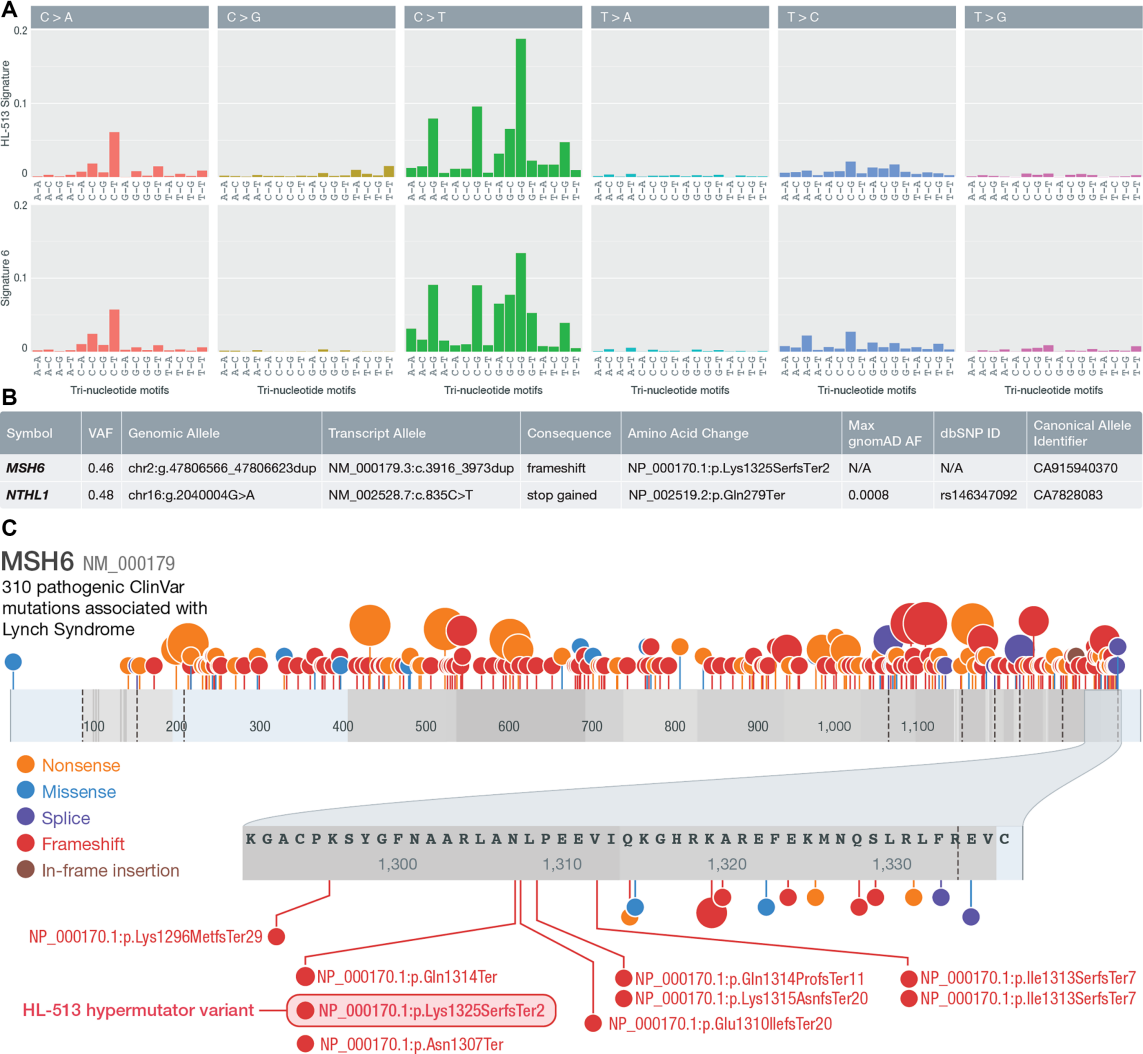
Hypermutated patient germline analysis. **A,** Comparison of trinucleotide sequence contexts of COSMIC signature 6 and HL-513 suggesting a high degree of similarity between the two. **B,** Summary of the MMR germline mutations identified in HL-513. **C,** Lolliplot of *MSH6* with Lynch syndrome mutations from ClinVar and the HL-513 mutation identified here. ClinVar mutations annotated as pathogenic and associated with Lynch syndrome are shown. The shaded oval highlights the duplication identified in HL-513. Note: mutations are plotted by ProteinPaint (103) according to (left-shifted) genomic coordinates but labeled with their HGVS expressions (right-shifted) from ClinVar explaining the discrepancies in some amino acid positions displayed.

To address the etiology of the hypermutated patient, the patient's germline was analyzed. Because we observed COSMIC mutation signature 6 in this patient, which is generally associated with defects in DNA mismatch repair (MMR) and microsatellite instability (MSI), an analysis for unique germline mutations in mismatch or base excision repair genes was performed (Fig. 7A; Supplementary Materials and Methods; Supplementary Table S12). Germline mutations in *NTHL1* and *MSH6* were discovered (Fig. 7B). The *NTHL1* mutation is an SNV in exon 1 causing a stop gain at Q287*. The *MSH6* mutation was a 58 bp duplication in exon 9, resulting in a frameshift (NM_000179; NP_000170.1:p.Lys1325SerfsTer2). This mutation appears the most likely candidate responsible for the hypermutated phenotype. This duplication is similar in kind and location to several frameshift and nonsense mutations reported in ClinVar that are known to cause Lynch syndrome, an autosomal dominant cancer predisposition syndrome characterized by MMR deficiency and MSI (Fig. 7C). This variant was also recently added to ClinVar (VCV001368701.1) and is interpreted to be pathogenic. We tested for the presence of MSI in our cohort and particularly in the hypermutated patient. The hypermutated patient had the highest percentage of mutated microsatellites of any sample and was the only sample above a threshold (3.5%) previously determined to be highly concordant with MSI-high status measured by IHC in colorectal cancer patients (Supplementary Table S13; ref. 72).

To confirm the unique nature of the germline mutations we identified in HL-513, we conducted a systematic analysis to identify germline MMR and base excision repair defects in the 29 non-hypermutated samples. These data were filtered as described in the supplementary methods. Excluding the results already described for HL-513, we identified 15 mutations in MMR/BER genes for 9 patients (Supplementary Table S13). These mutations were manually reviewed and are unlikely to be sequencing artifacts. Among these mutations, 14 were missense and one mutation was identified as a stop gain (*LIG3;ENST00000378526.8:c.73C>T*). The stop gain is not described in ClinVar. The scaled CADD (73) score for this variant is 31, suggesting that it is likely deleterious, but the ALoFT score (74) provided in the OpenCRAVAT report (75) suggests that this variant is only likely to cause a deleterious effect in the homozygous state. The VAF for this variant in the patient in which it occurred (HL-517) is 0.48, suggesting that it is heterozygous and therefore it is less likely to have a deleterious effect. This mutation is unlike the HL-513 duplication, which was recently added to ClinVar (VCV001368701.1) and was interpreted to be pathogenic. The missense variants identified are unlikely to cause a deleterious impact. Ten of the 14 variants were identified in ClinVar with interpretations of uncertain significance, benign, and likely benign. The three missense mutations not in ClinVar will require further interpretation, but the lack of hypermutated phenotype, would suggest that they are unlikely to cause a MMR or base excision repair phenotype. In summary, none of the mutations in the non-hypermutated samples were interpreted to have a pathogenic impact.

## Discussion

This study demonstrated the utility of ultra-deep sequencing to uncover recurrently mutated genes in low-frequency malignant cells and to further define the landscape of somatic mutations in cHL. We generated approximately 1,000x exomes in a cohort of 31 primarily newly diagnosed cHLs and matched nonmalignant germline tissues. Our somatic variants were validated with orthogonal error-corrected sequencing with a recovery rate >95%. Ultra-deep sequencing strategies have been employed to detect low VAF and/or subclonal variants in ovarian cancer (76), CLL (77), and AML (78, 79). However, here we pioneered this approach to overcome the progress-limiting rarity of HRS cells in cHL. Ultra-deep sequencing of cHL bulk biopsies confirmed the importance of previously reported pathways in cHL including JAK/STAT (Fig. 5), NFκB, and those relevant for immune evasion. Moreover, significantly recurrent mutations in previously unreported genes (*AXDND1*, *CDH5*, *LIMD2*, *OR13C2*, *PCDH7*, *RDH12*, *SCN9A*, and *STRAP*) and pathways (i.e., Hippo/YAP and MAPK) not often associated with cHL were discovered. Further confirmatory experiments are necessary to validate the relevance of these findings to cHL. Mutations associated with off-target AID activity that may be driving the pathogenesis of cHL were also revealed (Fig. 4). Furthermore, we used snRNA-seq to further validate that the variants identified here are likely to be from malignant cells. snRNA-seq data from one sample identified a cluster of cells with an exome-called somatic variant. This cluster of cells also showed evidence of an expanded B-cell clonotype, and these cells have differentially expressed genes that are consistent with known cHL biology. Finally, we identified a hypermutated patient with cHL and discovered a mutation in *MSH6* that is similar in kind and location to other variants associated with Lynch syndrome, which could be driving the patient's hypermutated phenotype (Fig. 7).

This study identified both known and novel mutations in cHL, with several previously reported JAK/STAT pathway mutations confirmed, including *STAT6*. Truncating mutations in, and proximal to, the ITIM located at the C-terminus of IL4R were discovered, revealing a novel potential mechanism for constitutive activation of STAT6 in cHL, by eliminating this suppressive function of IL4R signaling. It should be noted that IL4R mutations have been observed in Hodgkin lymphoma previously (80), but were not discussed in the context of STAT6 activation. Kashiwada and colleagues (81) found hyperproliferation in response to IL4 stimulation when the ITIM in IL4R is disrupted through site-directed mutagenesis in a murine bone marrow cell line, and this response was correlated with increased activation of STAT6. Mutations in IL4R in patients with cHL have been recently presented by Alig and colleagues and limited functional results provide preliminary confirmation of the relevance of our mutations to cHL.

The most recurrently mutated gene we uncovered is *SOCS1*, a finding observed by others (4, 7), suggesting concordance of our ultra-deep bulk exome approach with HRS purification approaches. This included several frameshift and nonsense mutations, as well as missense mutations, consistent with loss-of-function. SOCS proteins negatively regulate the JAK/STAT pathway, with loss-of-function leading to augmented JAK/STAT growth signaling. Several mutations were identified in the AID sequence recognition motif. Indeed, at *SOCS1* and *IGLL5,* 63% and 40%, respectively, of the SNVs we identified are potentially the result of off-target AID activity. It has been suggested that off-target canonical AID activity may be responsible for the pathogenesis of some lymphomas and leukemias (82–84). Mottok and colleagues have implicated aberrant AID activity resulting in off-target somatic hypermutation as the mechanism of *SOCS1* mutations in multiple germinal center lymphomas (85, 86). In addition to AID mutations in *SOCS1* and *IGLL5,* we also show that potentially aberrant AID mutations are present in *ARID5B*, *ZFP36L1,* and *HIST1H1B*. The results of our study suggest aberrant off-target AID activity is a characteristic of cHL and impacts several loci including *SOCS1*, which is the gene most impacted by recurrent somatic mutations in our cohort. Some of the mutation signatures we identified are similar to previous reports. For example, our mutation signature analysis showed the presence of noncanonical AID, which is consistent with other lymphoproliferative diseases (i.e., CLL; ref. 46). However, it is important to note that our mutation signature analysis did not identify any APOBEC mutation signatures, which was observed previously (7, 47) and our mutation signature analysis also did not recover evidence of canonical AID activity. This difference could be attributed to our small sample size or technical difference in the methods used to identify mutation signatures here compared with other studies. Previous studies (7, 47) used custom algorithms that can identify *de novo* mutation signatures. The methods we employed cannot identify new signatures. This difference likely limited our ability to identify the full spectrum of mutation signatures present among patients with cHL.

Mutations in 31 genes involved in Hippo regulation were identified, with 40% of our cohort (12/30) having at least one mutation in a Hippo regulating gene. A number of studies have suggested that human tumors use YAP/TAZ, which are integral transcriptional coactivators within the Hippo pathway, to facilitate proliferation, progression, migration, and metastasis (87). High expression of YAP was shown to be significantly correlated with disease progression and poor prognosis in DLBCL and knockdown of YAP expression suppressed cell proliferation in DLBCL cell lines (88). Furthermore, the overexpression of MST1 or the knockdown of YAP inhibited cell proliferation, promoted cell-cycle arrest, and apoptosis in natural killer/T-cell lymphoma (89). The most prominent mutated genes we identified that impact Hippo signaling are *CDH5* and *GNA13*, both of which were shown to be SMGs. CDH5, like many other cadherins, is involved in the adherens junction (AJ) that plays a role in cell architecture and Hippo pathway regulation. We identified a cluster of *CDH5* mutations that are in amino acids involved in CDH5/VE-cadherin's interaction with p120, beta-catenin, and plakoglobin. These associations are crucial for the stability of the AJ, which if disrupted could facilitate nuclear localization of YAP and TAZ, and in effect contribute to cell proliferation. All the mutations we identified in *CDH5* are missense, further work is needed to understand the functional impact of these mutations, but the clustered nature of the mutations suggests that this region is being targeted in cHL. In addition, the comparison of VAF distributions of the variants in this pathway to the JAK/STAT pathway suggests that variants in Hippo/TAZ/YAP regulating pathway are consistent with well-established pathways associated with cHL. The potential Hippo regulatory mutations we have presented here are hypothesis generating, further studies are necessary to address functional implications.

Among the other novel SMGs that were identified there is some literature to suggest that these genes are involved in the development of cancer or malignant phenotypes. For example, *RDH12* has been shown to be differentially expressed in cervical cancer and *RDH12* expression was negatively associated with tumor size and depth of cervical invasion (90)*.* In addition, *SCN9A* was shown to possibly promote gastric cancer progression (91). The novel SMGs we have identified are hypothesis generating and warrant further study in larger cohorts.

In addition to describing the landscape of somatic mutations in cHL we have demonstrated for the first time the potential of snRNA-seq technology in cHL. Identifying somatic mutations from single-cell data is very challenging. The inherent end bias, variable transcript abundance, and low number of reads per cell are among other challenges that make this a “needle-in-the-haystack” problem. Given the modest mutation burden and low tumor cellularity of cHL, it was not certain that any expressed variants would be discovered in the snRNA-seq data. However, in a single pilot sample, we successfully identified one somatic variant that we used to tag a cluster of presumed malignant cells. Our presumed cluster of malignant cells is further supported by the presence of an expanded BCR clonotype. Indeed, the coincident occurrence of a somatic mutation, with an expanded BCR clonotype is highly suggestive that we have identified a cluster of HRS cells. When we asked whether the presumed HRS cluster shows a pattern of gene expression that is distinct from other B cells we identified several differentially expressed genes that are consistent with cHL biology. Among the most upregulated genes in the presumed HRS cluster are *PIM1* and *PIM3*. It has been shown (92) that PIM1/2/3 are ubiquitously expressed in primary and cultured RS cells and are induced by JAK-STAT and NFκB pathways, two pathways known to be activated in cHL. We also observed upregulation of CD*83*, which was also identified as an SMG. Li and colleagues (2018; ref. 93) demonstrated that *CD83* is highly expressed in Hodgkin lymphoma cells lines and cells extracted from primary samples. Further work is needed to understand the expression patterns of *RASSF6* and *PTPRG*, which are generally considered to be tumor suppressors (94, 95) and genes like *BIRC3* and *CDKN1A* that are known to be involved in a number of different cancers (96, 97). Our analysis also identified some differentially expressed genes that are consistent with previous microarray-based studies including upregulation of *CDKN1A*, *CCR7*, and *NFKBIA*; and downregulation of *MS4A1*, *STAG3*, and *SSBP*. Our snRNA preliminary data point to the veracity of the somatic variants we identified and to the feasibility of snRNA-seq data to explore the functional consequences of somatic variants in HRS cells, interactions between HRS cells and the immune microenvironment, and the transcriptional signatures of cHL.

A single patient contributed over 70% of the overall somatic mutation burden in this cohort. Because hypermutated phenotypes are often a result of germline predisposition (36), we searched for a germline variant that may be responsible for this phenotype. Two mutations were identified, one SNV in *NTHL1* and a 58-base duplication in *MSH6*. Inactivating mutations in *MSH6*, a DNA MMR gene, are associated with Lynch syndrome. The frameshift variant we identified in *MSH6* is strikingly similar to other pathogenic variants known to cause Lynch syndrome. This patient also has a personal history of precancerous colon polyps and multiple benign breast masses. In addition, there is a family history that includes a sibling diagnosed with endometrial cancer. Unlike cHL, endometrial cancer has been strongly associated with Lynch syndrome and variants in *MSH6*. Endometrial cancer has a prevalence of 3% in patients with Lynch syndrome and is the most frequently observed cancer in women harboring pathogenic *MSH6* variants, with a 41% risk of developing endometrial cancer by age 70 (98, 99). Although cHL is not a cancer that is generally associated with Lynch syndrome, Wienand and colleagues (7) also identified two hypermutated cases in a cohort of patients with cHL. Wienand and colleagues (7) indicate that the hypermutator phenotypes they observed were associated with mutation signatures that are consistent with MSI (COSMIC signatures 6 and 15), which is similar to our result. They also report somatic alterations in *MSH3*, *MSH2,* and *ARID1A*, but do not report an analysis of germline variants. Our results confirm that hypermutation does occur in patients with cHL and suggest that this phenotype may be the result of germline cancer predisposition variants and could be important to guide therapy with immune checkpoint blockade. It is important to note that our hypermutated patient is a relapse patient. It is possible the observed mutation burden could be the result of prior chemotherapeutic treatments. However, the results of our analysis are consistent with a germline etiology of the hypermutation status.

There are several important limitations to our investigation of bulk cHL lymph node biopsies. A small percentage of variants (3.3%) discovered in the exomes were found to be false positives. These variants had a VAF that ranged from 0.50% to 13.53% and had a median of VAF 2.37%. This indicates that although there were some false positives (type I error) within our exomes, these false positives were not exclusively low VAF variants. We also show that these variants did not exclusively suffer from low exome coverage. Indeed, we show a nonsignificant difference in the level of coverage between sites that passed and failed validation (Supplementary Fig. S6). However, we did observe a relationship between median VAF and the number of variants that were found to be true positives. Several samples were characterized by a relatively small number of variant calls, with lower median VAF, and higher false positive rate. However, this did not prevent us from validating as many as approximately 25 sites in samples with median VAFs as low as 1.5%. This suggests that although deep exome sequencing can reliably uncover a spectrum of somatic variation in HRS cells, we were not able to fully overcome the limits of low tumor cellularity in some cases. This challenge may in part explain why our median mutation burden is lower than others have reported (8). We identified a mean of 32 variants per patient, excluding the hypermutated patient, which is similar to the median number of variants identified by Tiacci and colleagues (2018). But our median number of mutations (11 mutations) is smaller than their median number of mutations observed previously. However, 13/30 (43%) of the patients in our cohort have >20 variants, suggesting that many of the samples we sequenced have a mutation burden that is comparable to what was observed by Tiacci and colleagues (2018). As a further point of comparison, our mean number of variants is smaller than what is reported by Wienand and colleagues (7); however, it is not indicated whether the hypermutated patients they uncovered were excluded in their calculation of the mean number of variants. If we calculated our mean including the hypermutated sample then our mean would be 137 variants, which is similar to what was observed by Wienand and colleagues (7). Larger cohorts will help to eliminate sampling bias and will assist in clarifying the typical mutation burden of cHL.

Another limitation of this study is that we do not have a clear false negative rate (type II error) and it is likely that in some samples with lower tumor cellularity we failed to identify some somatic mutations in the original exome pipeline. However, our HaloPlex experiment helped to illuminate some missed variants and helped increase our sensitivity. As we continue to explore the use of deep sequencing to uncover somatic variants, we will adjust our pipeline to correct the procedures that caused these variants to be missed. Low tumor cellularity is a challenge for all approaches and typically leads to some samples being excluded entirely (e.g., with insufficient output from flow sorting might preclude sequencing altogether). We did not exclude any samples based on cellularity estimates.

Finally, while we achieved an average of 1,000x depth of exome coverage, our coverage, like most exome sequencing, is not uniformly distributed. Approximately one-third of the bases in the tumor and normal samples achieved less than 400x coverage. These regions of lesser coverage may also be prone to false negatives. However, we confirmed recurrently mutated genes previously reported in studies that included enrichment for HRS cells, including known hotspot mutations and mutations with predicted consequences that are consistent with known Hodgkin lymphoma biology (e.g., inactivating mutations in *SOCS-1* and *TNFAIP3*). Furthermore, 22 individuals (73% of our cohort) have at least one mutation in a gene that has been suggested to drive cHL pathogenesis or the pathogenesis of another lymphoma (7–10). The variants in these genes have an average VAF of 4.4%, which is consistent with variants from an HRS cell. In addition, the average depth of coverage at known cHL genes is 1,353 reads, suggesting we have sufficient coverage to call variants at this low VAF. Together these data suggest that while our coverage was not uniform, we positively identified variants in genes known to be mutated in cHL and this trend was consistent across most of the cohort. In addition, because our analysis identified many previously described mutation patterns attributed to HRS cells, the novel genes, and variants we identified are also likely to characterize HRS cells.

A recent study of clonal hematopoiesis of indeterminate potential (CHIP) in microdissected HRS cells reported that 5/40 patients diagnosed with Hodgkin lymphoma had mutations consistent with CHIP in the microenvironment, or, as in one case, in HRS cells (100). These data suggest that CHIP has the potential to obscure our ability to detect somatic variants from bulk cHL lymph node biopsies. To address this concern, we interrogated our data for a pattern of recurrent mutation in genes associated with CHIP described in Husby and colleagues (2020; ref. 101) and Niroula and colleagues (2021; ref. 102). Our analysis did not detect a pattern of recurrent mutations in genes associated with CHIP (101, 102). Furthermore, given that the median age of our cohort is approximately 36 years old, we suggest that the somatic mutations we identified are unlikely to be the result of a contaminating signal from CHIP. In the work by Niroula and colleagues (2021), the authors propose the concept of “L-CHIP,” in which clonal hematopoiesis is defined by mutations in common lymphoid malignancy driver genes. They suggest that patients with L-CHIP are at an increased risk of subsequently developing lymphoid malignancies, in contrast with the classical myeloid malignancy risk attributed to traditional CHIP (or “M-CHIP” in the work by Niroula and colleagues). Potential overlap between our set of genes somatically mutated in cHL and the set of genes mutated in L-CHIP has little impact on our conclusions about the somatic mutations identified in our study. If we had looked in the peripheral blood for CHIP in our Hodgkin lymphoma cohort and found L-CHIP associated with the somatic variants identified here we would conclude that some patients in our cohort have circulating precursor lesions.

In summary, we have shown that ultra-deep sequencing can be used to identify somatic variants in rare malignant HRS cells. We have further demonstrated using snRNA-seq data that the somatic variants we identified can be used to identify a cluster of cells that are likely to be HRS cells. This proof-of-concept opens the possibility of careful dissection of the transcriptome and cell–cell interactions that characterize cHL. We have further described the role that aberrant somatic hypermutation plays in cHL, and we have suggested a novel role of mutations in IL4R in the constitutive activation of STAT6 that deserves further examination. We also revealed that genes regulating Hippo signaling may play a role in cHL. This work demonstrates the utility of ultra-deep sequencing in bulk cHL lymph node biopsies as an alternative to laborious cell isolation techniques. Previous work has demonstrated the utility of ct-DNA in the setting of Hodgkin lymphoma, and we acknowledge this source of DNA remains a viable option for clinical approaches. However, as sequencing costs continue to decrease, the methods we employed here provide a platform to attempt larger cohort sequencing of genomic DNA from primary cHL samples. This is especially true when capture costs are minimized. With the continued development of UMI-based sequencing methodologies, deep sequencing methods open new avenues of research facilitating a practical approach to identifying and correlating cHL mutations with clinical outcomes.

## Supplementary Material

Supplementary Methods supplemental methods

Supplementary Figure 1 Exome VAF and Depth of Coverage

Supplementary Figure 2 Exome VAF and HaloPlex Tumor VAF

Supplementary Figure 3 Relationship between Variant Validation Rate and The Number of HRS Cells/HPF

Supplementary Figure 4 Exome Tumor VAF Distribution Partitioned by Validation Status

Supplementary Figure 5 Exome Tumor VAF Distribution and Exome Tumor Coverage Partitioned by Validation Status

Supplementary Figure 6 Coverage Summary Across Sites that Passed and Failed Validation

Supplementary Figure 7 figure 7Relationship between median Tumor VAF and Total Variant Count by Sample

Supplementary Figure 8 Comparison of Genes Shown to be Recurrently Mutated in Studies of Adult cHL

Supplementary Figure 9 Co-Occurrence and Mutual Exclusivity Among Significantly Mutated Genes

Supplementary Figure 10 Comparison of mutation burden and EBV status

Supplementary Figure 11 Summary of Recurrently Mutated Genes for Relapsed Samples

Supplementary Figure 12 Normalized Expression of EIF4A2

Supplementary Figure 13 HL-248 Reads Covering EIF4A2 in Cluster 14

Supplementary Figure 14 Observed COSMIC v.3 mutation signatures

Supplementary Figure 15 VAF Distribution of JAK/STAT and Hippo/TAZ/YAP regulation associated variants

Supplementary Figure 16 Diagram of observed mutated components of the MAPK signaling cascade

Supplementary Figure 17 Diagram of observed mutated components of the PI3K signaling cascade

Supplementary Tables 1-14 Supplemental Tables 1-14

Supplementary Data File 1 Haloplex amplicons
