# Supplementary material for: Ultra-Deep Sequencing Reveals the Mutational Landscape of Classical Hodgkin Lymphoma
Source: Cancer Res Commun. 2023 Nov 15;3(11):2312–30. doi: 10.1158/2767-9764.CRC-23-0140 (PMC10648575; doi:10.1158/2767-9764.CRC-23-0140)
Supplement: Supplementary Methods — supplemental methods [file crc-23-0140-s01.docx]

## **Supplementary methods for “Ultra Deep Sequencing Reveals the Mutational Landscape of Classical Hodgkin Lymphoma”**

Felicia Gomez^1,2,5^, Bryan Fisk^2^, Joshua McMichael^2^, Matthew Mosior^2^ ,Jennifer A. Foltz^1^, Zachary L. Skidmore^2^, Eric J. Duncavage^3^, Christopher A. Miller^1,2^, Haley J. Abel^1,2^,Yi-Shan Li^3^, David A. Russler-Germain^1^ , Kilannin Krysiak^2,3,5^, Marcus P. Watkins^1^, Cody Ramirez^2^, Alina Schmidt^2^, Fernanda Martins Rodrigues^2^, Lee Trani^2^, Ajay Khanna^1^, Julia A. Wagner^1^, Robert S. Fulton^2^, Catrina Fronick^2^, Michelle O’Laughlin^2^, Timothy Schappe,^1^ Amanda Cashen^1^, Neha Mehta-Shah^1^, Brad S. Kahl^1^, Jason Walker^2^, Nancy L. Bartlett^1^, Malachi Griffith^1,2,4,5*^, Todd A. Fehniger^1,5*^, Obi L. Griffith^1,2,4,5*^

1. Department of Medicine, Division of Oncology, Washington University School of Medicine, St Louis, MO

2. McDonnell Genome Institute, Department of Medicine, Washington University School of Medicine, St Louis, MO.

3. Department of Pathology and Immunology, Washington University School of Medicine, St Louis, MO.

4. Department of Genetics, Washington University School of Medicine, St Louis, MO

5. Siteman Cancer Center, Washington University School of Medicine, St Louis, MO.

***Correspondence:

Obi L. Griffith (obigriffith@wustl.edu), Todd A Fehniger (tfehnige@wustl.edu), and Malachi Griffith (mgriffit@wustl.edu)

##

##

##

##

##

## **Supplementary Methods**

### *Sequence alignment, variant calling, and filtering*

Sequence analysis and data management was performed using the Genome Modeling System (GMS)[(1)](https://paperpile.com/c/N3ua4R/Bw7NL). Briefly, paired-end reads were aligned to human reference sequence GRCh38 using BWA-MEM v0.7.10-r789 [(2)](https://paperpile.com/c/N3ua4R/1Yifh) via the Speedseq analysis platform (v0.10)[(3)](https://paperpile.com/c/N3ua4R/6JCi0). Duplicate marking was completed using samblaster v0.1.22[(4)](https://paperpile.com/c/N3ua4R/nol2q) and alignments were sorted using sambamba v0.5.4[(5)](https://paperpile.com/c/N3ua4R/ByVmE). Somatic variants were called using SAMtools[(6)](https://paperpile.com/c/N3ua4R/pORJ6), SomaticSniper[(7)](https://paperpile.com/c/N3ua4R/AGzl2), VarScan[(8)](https://paperpile.com/c/N3ua4R/k5ZTR), MuTect[(9)](https://paperpile.com/c/N3ua4R/ZiLgZ), Strelka[(10)](https://paperpile.com/c/N3ua4R/APRDh), Pindel[(11)](https://paperpile.com/c/N3ua4R/PsgWi), and GATK[(12)](https://paperpile.com/c/N3ua4R/79QGj). Variants were required to be called by at least one variant caller.

Following variant calling, all SNVs and INDELs were annotated using an in-house annotation pipeline[(1)](https://paperpile.com/c/N3ua4R/Bw7NL). After variant annotation, all variants were filtered to remove common variants, sequencing errors, and pipeline artifacts. These filters included several automated filters that were applied to our data, that are described as follows; we required all variants to have a minimum of 50x depth in both the tumor and normal samples. This filter was assessed on coverage achieved from the combination of all three libraries. We excluded sites with the following characteristics: normal VAF >= 5%, tumor VAF <= 0.5% and sites with <= 5 variant supporting reads in the tumor. We filtered sites based on their predicted consequences. We removed sites from the 5’ and 3’ UTRs, intronic sites, synonymous variants, and sites annotated to non-coding transcripts. To remove artifacts and sequencing errors generated in this dataset by the pipelines, after somatic variant calling was performed, we also counted the number of variant supporting reads in the tumor samples with the --per-library setting using bam-readcount[(13)](https://paperpile.com/c/N3ua4R/XpPJK). Within each library a variant was required to have at least 3 reads of support and a VAF >0.5%. As a further attempt to remove pipeline artifacts and sequencing errors, all variants were filtered against the 31 normal samples from this study. In this step, SNVs were removed if they had >4 variant reads and >1% VAF in 2 or more normal samples and INDELs removed if they had >3 variant reads and >1% VAF in 3 or more normal samples. Variants were removed if their global minor allele frequency was >0.001 in ExAC release 0.2[(14)](https://paperpile.com/c/N3ua4R/jMYG6).

Following all automated filters, experienced analysts reviewed all variants in IGV[(15)](https://paperpile.com/c/N3ua4R/W4yNl) using published manual review standard operating procedures[(16)](https://paperpile.com/c/N3ua4R/DElrC) to identify sequencing errors, variant caller artifacts, false positives (e.g., germline variants) and other systematic anomalies.

*snRNA Alignment and Analysis*

### Using Cell Ranger 3.1.0 (10x Genomics) fastq files were aligned to the build38 pre-mRNA reference provided by 10x Genomics (this allows mapping to both exons and introns). Reads were counted using 10x Genomics Cell Ranger. Following alignment, the Seurat R package (version 4.1.1)[(17)](https://paperpile.com/c/N3ua4R/8Kn3g) was used for data normalization, batch-correction, clustering, visualization, and differential expression analysis. After the cell expression matrix from Cell Ranger was read into Seurat, nuclei that expressed less than 100 features were removed as well as genes that were expressed in less than 10 nuclei. Nuclei with a proportion of mitochondrial genes greater than the 95th percentile were removed. The expression matrix was then normalized by taking each gene count and dividing it by the total gene counts in the nucleus, then each normalized value was multiplied by a scaling factor of 10,000 and natural log transformed. Variable genes were selected by Seurat’s implementation of the variance-stabilizing transformation function with a loess_span of 0.3 and clip_max set to auto. The gene values were then scaled by dividing by their root mean square. Principal component analysis was performed on the variable genes and dimensionality reduction was performed using Seurat’s implementation of the UMAP algorithm. Doublets were identified using the R package DoubletFinder[(18)](https://paperpile.com/c/N3ua4R/RsPGz) following the example provided here:<https://github.com/chris-mcginnis-ucsf/DoubletFinder> with the exception that the expected doublet rate was interpolated from the table in the Chromium Next GEM Single Cell 5’ Reagent Kits v2 protocol. In addition to unsupervised clustering of nuclei using the UMAP algorithm, nuclei were also assigned to cell types using the R package SingleR with the reference expression profiles derived from Monaco et al. (2019)[(19)](https://paperpile.com/c/N3ua4R/CL26b) and obtained from the celldex SingleR package. Vartrix (10x Genomics) was used to associate cell barcodes with variant supporting reads and to count the number of variant supporting reads present in each nucleus. Vartrix was run in coverage (--scoring-method coverage) and umi-aware (--umi) modes.

### The B-cell receptor V(D)J library was processed using Cell Ranger VDJ. Fastq files obtained from V(D)J libraries were aligned to the GRCh38 V(D)J reference provided by 10x Genomics using the 10x Genomics Cell Ranger VDJ 3.1.0 pipeline. Following the identification of a dominant clonotype, any clonotype that was one amino acid different from the dominant clonotype was assumed to be derived from the dominant clonotype. These clonotypes were grouped with the dominant clonotype. Nuclei expressing the dominant clonotypes were identified and are predominantly expressed in clusters 3 and 14. To identify the B-cell cluster(s) that cluster 14 is most similar to, the following analysis was undertaken: First we identified the top 2000 variable genes in all cells using the variance stabilizing transformation (VST) implementation in Seurat. Then in each B-cell cluster that was identified as having at least 50% B-cells (clusters 0, 1, 3, 4, 9, 12) the average expression of each of these 2000 genes was found across all B-cells in that cluster. The average expression profile from cluster 14 was then compared against the other clusters’ average expression profile using a Pearson correlation and we assessed similarity based on the strength of the expression pattern correlation. Several differential gene expression analyses were performed using the Seurat FindMarkers function. A pairwise comparison between all clusters identified as B-cells was performed as well as a comparison between cluster 3 and all other clusters identified as B-cells. In each case ribosomal and mitochondrial genes were removed, as well as genes only expressed in one nucleus.

To determine whether the other variants discovered in our deep exomes could have been identified in an snRNA experiment similar to the experiment presented here if all samples were included, we used bam readcount[(13)](https://paperpile.com/c/N3ua4R/XpPJK) to assess the read support for all other discovered variants in a bam file only containing reads with barcodes annotated to cluster 3. We then assessed the number of reads covering each variable position. A position with at least 2000 reads was determined to be a variable position with a high likelihood of detection if expressed.

### *Hotspot variant read counting*

To interrogate our data for known hotspots in cHL, we used bam-readcount (<https://github.com/genome/bam-readcount>) on 10 known variants (6 variants in *STAT6*, 3 variants in *XPO1* and 1 variant in *NFKBIE* - Supplemental Table 1) in all consensus tumor and normal bams. Sites were required to have a tumor VAF >= 0.5%, normal VAF < 5%, a tumor var count > 5 reads, and a normal var count <= 2 reads. We also required a total of 5 reads in the tumor and normal. Additionally, similar to the consensus *de novo* variant calling, all variants that passed the automated filters were manually reviewed using both consensus bams and the exome bams. All hot spot variants were required to have read support in both the exome and haloplex tumor bams.

Following all variant calling (initial and *de novo*), variant filtering, and manual review, as an additional quality control verification we combined all data for all variants reported in this manuscript (exome and HaloPlex error-corrected reads) to verify that all variants do not have more support in the normal than the tumor. There were no variants for which there was more support in the normal than the tumor. The mean difference between the combined variant support in the tumor and normal (where a negative value would indicate more support in the normal than the tumor) is 293.23 (median=225.5), suggesting that most of the variants described in our manuscript are highly tumor specific.

***Variant Analyses***

### *Significantly mutated gene analysis*

### We identified significantly mutated genes (SMGs) using MuSiC (both with and without HL-513)[(20)](https://paperpile.com/c/N3ua4R/RzK7p) and using dN/dScv (both with and without HL-513)[(21)](https://paperpile.com/c/N3ua4R/weRz7). For the MuSiC analysis, the region of interest was restricted to the coding space covered by the exome reagent. Additionally, two bases were added to the beginning and end of each exon to account for splice sites. All default parameters were used. Significance was determined by comparing the minimum FDR value from the convolution and likelihood ratio tests to our predetermined threshold - 0.05. For the dN/dScv analysis all default parameters were used with an hg38 covariates file retrieved from the dN/dScv supplemental data github repository (<https://github.com/im3sanger/dndscv_data>). The silent exome variants used in this analysis were processed through the automated VAF and coverage filters described in the exome data filtering section above.

#### *Pathway analysis*

To identify pathways that are impacted by our somatic mutations we first downloaded the C2 curated gene set collection from MSigDB[(22,23)](https://paperpile.com/c/N3ua4R/6UP2u+xnwfB). This gene set includes canonical pathways from several online databases including BioCarta, KEGG, and Reactome. This gene set also includes genes from the biomedical literature that have been shown to be related to important biological and clinical states (e.g., cancer metastasis or drug resistance). We also included a list of 1,600 known human transcription factors[(24)](https://paperpile.com/c/N3ua4R/ucHTr), a list of known regions of off-target AID activity in DLBCL and FL,[(25,26)](https://paperpile.com/c/N3ua4R/uVOB2+44nxV) and selected WikiPathways[(27)](https://paperpile.com/c/N3ua4R/WUOYS) (e.g. Hippo regulating pathways). Note that the MAPK signaling pathway was defined as the genes included in the KEGG MAPK signaling pathway with the addition of the following genes: *PCDH7*[*(28)*](https://paperpile.com/c/N3ua4R/T2acC)*, TRAF7*[*(29)*](https://paperpile.com/c/N3ua4R/7cy6U), *ARAF, DAB2IP, SHC3, IRS2, KSR2,* and *KSR1*[*(30)*](https://paperpile.com/c/N3ua4R/KhinL). We identified the pathways from these gene sets that were impacted by our most recurrently mutated genes by searching for genes mutated in 3 or more patients. When a recurrently mutated gene was mapped to more than one pathway, manual inspection was used to retain the pathway(s) that have been shown in previous reports to contain genes that are mutated in lymphoma(s). This criterion was employed to manage the number of pathways that were elevated for further analysis. Following the creation of our “pathways of interest” data set, we further annotated all genes mutated in all patients to these pathways.

*Activation Induced Deaminase Analysis*

In the genes we identified as potential targets of aberrant AID activity in the pathway analysis described above[(25,26)](https://paperpile.com/c/N3ua4R/44nxV+uVOB2) (Supplementary Table 4) we tested whether these loci contain somatic mutations (SNVs) that are likely to be the product of aberrant AID activity. SNVs were considered products of off target AID activity if they met the following criteria: the SNV must have a C (or G) as the reference allele, be within 2 Kb of transcript start sites (TSS) and be within the WRC/GYW AID sequence motif. To identify whether a mutation is within an AID sequence motif we used custom software (Fasta-Region-Inspector (FRI):<https://github.com/Matthew-Mosior/Fasta-Region-Inspector>).

To assess the significance of the number of AID target mutations we identified, we used additional custom software (Fasta-Region-Randomizer:<https://github.com/Matthew-Mosior/Fasta-Region-Randomizer>) to create 100,000 simulated datasets that contain the same number of SNVs observed within each target gene. In these simulations, the genomic space that was considered included all exons of each gene (i.e., the exonic space included in the exome capture). We then determined the observed number of putative AID mutations in each simulated dataset using FRI. To assess whether the overall number of off-target AID mutations is significantly different from random expectations we calculated how many times our total number of actual AID mutations (or greater) was observed in the simulated data.

*Mutation signature analysis*

Mutation signatures were assigned using the deconstructSigs R package.[(31)](https://paperpile.com/c/N3ua4R/Yq0yp) The reference signatures evaluated were the 96 COSMIC v3 signatures of mutational processes in human cancers (<http://cancer.sanger.ac.uk/cosmic/signatures>). Samples that contained >= 50 SNVs were included in this analysis.

#### *Germline analysis*

Sequencing data from the hypermutated patient was interrogated for germline variants within genes that are involved in mismatch repair and base excision repair (Supplemental Table 2). Sequence data was aligned using the same methods previously described for the WashU post harmonization alignment and data processing pipeline.[(32)](https://paperpile.com/c/N3ua4R/D0aau) Briefly, reads were aligned using BWA MEM v0.7.15-r1140[(2)](https://paperpile.com/c/N3ua4R/1Yifh), MC (mate CIGAR) and MQ (mate mapping quality) tags were added using samblaster v0.1.24[(4)](https://paperpile.com/c/N3ua4R/nol2q), alignments were sorted using sambamba (27), and duplicates were marked using Picard (<http://broadinstitute.github.io/picard/>). Variants were called using the GATK Haplotype caller (v3.5.0) and the GATK Genotype Caller v3.4-0-gf196186.[(33)](https://paperpile.com/c/N3ua4R/2A4SC)

Following variant identification, variants were annotated using VEP (Ensembl v93). To create a high-quality variant list, variants were required to have a depth of coverage ≧20 reads, ≧ 5 variant supporting reads, and a VAF ≧ 20%. Additionally, to remove variants less likely to impact gene function we removed variants annotated by VEP as located in the 5’ or 3’ UTR, synonymous, non-coding, downstream, upstream, nonsense mediated decay variants, and variants located in gene regions that have been annotated as regulatory regions. We also applied a gnomad (v2.1)[(34)](https://paperpile.com/c/N3ua4R/lAGbH) and gnomad (v3.1.2)[(35)](https://paperpile.com/c/N3ua4R/5LnB5) population allele frequency filter where we removed variants with a MAX_AF > 0.1%. Following all filters, the remaining variants were further examined through manual review[(15)](https://paperpile.com/c/N3ua4R/W4yNl).

To interrogate our cohort for the presence of microsatellite instability (MSI) we utilized MSIsensor Pro[(36)](https://paperpile.com/c/N3ua4R/2SJD7) with the default parameters. Following the original MSIsensor recommendations[(37)](https://paperpile.com/c/N3ua4R/gXEml) and an analysis of exome sequenced colorectal tumors that showed 3.5% as a highly reliable threshold for MSI high tumors[(38)](https://paperpile.com/c/N3ua4R/ePQWq), we used a 3.5% as the threshold for the identification of MSI in our cohort (Supplemental Table 7).

**References**

1. [Griffith M, Griffith OL, Smith SM, Ramu A, Callaway MB, Brummett AM, et al. Genome Modeling System: A Knowledge Management Platform for Genomics. PLoS Comput Biol. 2015;11:e1004274.](http://paperpile.com/b/N3ua4R/Bw7NL)

2. [Li H. Aligning sequence reads, clone sequences and assembly contigs with BWA-MEM. arXiv preprint arXiv:13033997 [Internet]. arxiv.org; 2013; Available from:](http://paperpile.com/b/N3ua4R/1Yifh) <http://arxiv.org/abs/1303.3997>

3. [Chiang C, Layer RM, Faust GG, Lindberg MR, Rose DB, Garrison EP, et al. SpeedSeq: ultra-fast personal genome analysis and interpretation. Nat Methods. 2015;12:966–8.](http://paperpile.com/b/N3ua4R/6JCi0)

4. [Faust GG, Hall IM. SAMBLASTER: fast duplicate marking and structural variant read extraction. Bioinformatics. 2014;30:2503–5.](http://paperpile.com/b/N3ua4R/nol2q)

5. [Artem Tarasov PP. sambamba [Internet]. 2012. Available from:](http://paperpile.com/b/N3ua4R/ByVmE) <http://lomereiter.github.io/sambamba/docs/sambamba-view.html>

6. [Li H, Handsaker B, Wysoker A, Fennell T, Ruan J, Homer N, et al. The Sequence Alignment/Map format and SAMtools. Bioinformatics. 2009;25:2078–9.](http://paperpile.com/b/N3ua4R/pORJ6)

7. [Larson DE, Harris CC, Chen K, Koboldt DC, Abbott TE, Dooling DJ, et al. SomaticSniper: identification of somatic point mutations in whole genome sequencing data. Bioinformatics. 2012;28:311–7.](http://paperpile.com/b/N3ua4R/AGzl2)

8. [Koboldt DC, Zhang Q, Larson DE, Shen D, McLellan MD, Lin L, et al. VarScan 2: somatic mutation and copy number alteration discovery in cancer by exome sequencing. Genome Res. 2012;22:568–76.](http://paperpile.com/b/N3ua4R/k5ZTR)

9. [Cibulskis K, Lawrence MS, Carter SL, Sivachenko A, Jaffe D, Sougnez C, et al. Sensitive detection of somatic point mutations in impure and heterogeneous cancer samples. Nat Biotechnol. 2013;31:213–9.](http://paperpile.com/b/N3ua4R/ZiLgZ)

10. [Saunders CT, Wong WSW, Swamy S, Becq J, Murray LJ, Cheetham RK. Strelka: accurate somatic small-variant calling from sequenced tumor-normal sample pairs. Bioinformatics. 2012;28:1811–7.](http://paperpile.com/b/N3ua4R/APRDh)

11. [Ye K, Schulz MH, Long Q, Apweiler R, Ning Z. Pindel: a pattern growth approach to detect break points of large deletions and medium sized insertions from paired-end short reads. Bioinformatics. 2009;25:2865–71.](http://paperpile.com/b/N3ua4R/PsgWi)

12. [McKenna A, Hanna M, Banks E, Sivachenko A, Cibulskis K, Kernytsky A, et al. The Genome Analysis Toolkit: a MapReduce framework for analyzing next-generation DNA sequencing data. Genome Res. 2010;20:1297–303.](http://paperpile.com/b/N3ua4R/79QGj)

13. [Khanna A, Larson DE, Srivatsan SN, Mosior M, Abbott TE, Kiwala S, et al. Bam-readcount -- rapid generation of basepair-resolution sequence metrics. ArXiv [Internet]. 2021; Available from:](http://paperpile.com/b/N3ua4R/XpPJK) <https://www.ncbi.nlm.nih.gov/pubmed/34341766>

14. [Lek M, Karczewski KJ, Minikel EV, Samocha KE, Banks E, Fennell T, et al. Analysis of protein-coding genetic variation in 60,706 humans. Nature. 2016;536:285–91.](http://paperpile.com/b/N3ua4R/jMYG6)

15. [Barnell EK, Ronning P, Campbell KM, Krysiak K, Ainscough BJ, Sheta LM, et al. Standard operating procedure for somatic variant refinement of sequencing data with paired tumor and normal samples [Internet]. Genetics in Medicine. 2019. page 972–81. Available from:](http://paperpile.com/b/N3ua4R/W4yNl) <http://dx.doi.org/10.1038/s41436-018-0278-z>

16. [Thorvaldsdóttir H, Robinson JT, Mesirov JP. Integrative Genomics Viewer (IGV): high-performance genomics data visualization and exploration. Brief Bioinform. 2013;14:178–92.](http://paperpile.com/b/N3ua4R/DElrC)

17. [Hao Y, Hao S, Andersen-Nissen E, Mauck WM 3rd, Zheng S, Butler A, et al. Integrated analysis of multimodal single-cell data. Cell. 2021;184:3573–87.e29.](http://paperpile.com/b/N3ua4R/8Kn3g)

18. [McGinnis CS, Murrow LM, Gartner ZJ. DoubletFinder: Doublet Detection in Single-Cell RNA Sequencing Data Using Artificial Nearest Neighbors. Cell Syst. 2019;8:329–37.e4.](http://paperpile.com/b/N3ua4R/RsPGz)

19. [Monaco G, Lee B, Xu W, Mustafah S, Hwang YY, Carré C, et al. RNA-Seq Signatures Normalized by mRNA Abundance Allow Absolute Deconvolution of Human Immune Cell Types. Cell Rep. 2019;26:1627–40.e7.](http://paperpile.com/b/N3ua4R/CL26b)

20. [Dees ND, Zhang Q, Kandoth C, Wendl MC, Schierding W, Koboldt DC, et al. MuSiC: identifying mutational significance in cancer genomes. Genome Res. 2012;22:1589–98.](http://paperpile.com/b/N3ua4R/RzK7p)

21. [Martincorena I, Raine KM, Gerstung M, Dawson KJ, Haase K, Van Loo P, et al. Universal Patterns of Selection in Cancer and Somatic Tissues. Cell. 2017;171:1029–41.e21.](http://paperpile.com/b/N3ua4R/weRz7)

22. [Subramanian A, Tamayo P, Mootha VK, Mukherjee S, Ebert BL, Gillette MA, et al. Gene set enrichment analysis: a knowledge-based approach for interpreting genome-wide expression profiles. Proc Natl Acad Sci U S A. 2005;102:15545–50.](http://paperpile.com/b/N3ua4R/6UP2u)

23. [Liberzon A, Subramanian A, Pinchback R, Thorvaldsdóttir H, Tamayo P, Mesirov JP. Molecular signatures database (MSigDB) 3.0. Bioinformatics. 2011;27:1739–40.](http://paperpile.com/b/N3ua4R/xnwfB)

24. [Lambert SA, Jolma A, Campitelli LF, Das PK, Yin Y, Albu M, et al. The Human Transcription Factors. Cell. 2018;175:598–9.](http://paperpile.com/b/N3ua4R/ucHTr)

25. [Khodabakhshi AH, Morin RD, Fejes AP, Mungall AJ, Mungall KL, Bolger-Munro M, et al. Recurrent targets of aberrant somatic hypermutation in lymphoma. Oncotarget. 2012;3:1308–19.](http://paperpile.com/b/N3ua4R/uVOB2)

26. [Okosun J, Bödör C, Wang J, Araf S, Yang C-Y, Pan C, et al. Integrated genomic analysis identifies recurrent mutations and evolution patterns driving the initiation and progression of follicular lymphoma. Nat Genet. 2014;46:176–81.](http://paperpile.com/b/N3ua4R/44nxV)

27. [Martens M, Ammar A, Riutta A, Waagmeester A, Slenter DN, Hanspers K, et al. WikiPathways: connecting communities. Nucleic Acids Res. 2021;49:D613–21.](http://paperpile.com/b/N3ua4R/WUOYS)

28. [Zhou X, Updegraff BL, Guo Y, Peyton M, Girard L, Larsen JE, et al. PROTOCADHERIN 7 Acts through SET and PP2A to Potentiate MAPK Signaling by EGFR and KRAS during Lung Tumorigenesis. Cancer Res. 2017;77:187–97.](http://paperpile.com/b/N3ua4R/T2acC)

29. [Zotti T, Scudiero I, Vito P, Stilo R. The Emerging Role of TRAF7 in Tumor Development. J Cell Physiol. 2017;232:1233–8.](http://paperpile.com/b/N3ua4R/7cy6U)

30. [Jassal B, Matthews L, Viteri G, Gong C, Lorente P, Fabregat A, et al. The reactome pathway knowledgebase. Nucleic Acids Res. 2020;48:D498–503.](http://paperpile.com/b/N3ua4R/KhinL)

31. [Rosenthal R, McGranahan N, Herrero J, Taylor BS, Swanton C. DeconstructSigs: delineating mutational processes in single tumors distinguishes DNA repair deficiencies and patterns of carcinoma evolution. Genome Biol. 2016;17:31.](http://paperpile.com/b/N3ua4R/Yq0yp)

32. [Regier AA, Farjoun Y, Larson D, Krasheninina O, Kang HM, Howrigan DP, et al. Functional equivalence of genome sequencing analysis pipelines enables harmonized variant calling across human genetics projects [Internet]. Available from:](http://paperpile.com/b/N3ua4R/D0aau) <http://dx.doi.org/10.1101/269316>

33. [Poplin R, Ruano-Rubio V, DePristo MA, Fennell TJ, Carneiro MO, Van der Auwera GA, et al. Scaling accurate genetic variant discovery to tens of thousands of samples [Internet]. 2017 [cited 2020 Oct 19]. page 201178. Available from:](http://paperpile.com/b/N3ua4R/2A4SC) <https://www.biorxiv.org/content/10.1101/201178v2.abstract>

34. [Karczewski KJ, Francioli LC, Tiao G, Cummings BB, Alföldi J, Wang Q, et al. The mutational constraint spectrum quantified from variation in 141,456 humans. Nature. 2020;581:434–43.](http://paperpile.com/b/N3ua4R/lAGbH)

35. [Chen S, Francioli LC, Goodrich JK, Collins RL, Wang Q, Alföldi J, et al. A genome-wide mutational constraint map quantified from variation in 76,156 human genomes [Internet]. bioRxiv. 2022 [cited 2022 Oct 13]. page 2022.03.20.485034. Available from:](http://paperpile.com/b/N3ua4R/5LnB5) <https://www.biorxiv.org/content/biorxiv/early/2022/03/21/2022.03.20.485034>

36. [Jia P, Yang X, Guo L, Liu B, Lin J, Liang H, et al. MSIsensor-pro: Fast, Accurate, and Matched-normal-sample-free Detection of Microsatellite Instability. Genomics Proteomics Bioinformatics. 2020;18:65–71.](http://paperpile.com/b/N3ua4R/2SJD7)

37. [Niu B, Ye K, Zhang Q, Lu C, Xie M, McLellan MD, et al. MSIsensor: microsatellite instability detection using paired tumor-normal sequence data. Bioinformatics. 2014;30:1015–6.](http://paperpile.com/b/N3ua4R/gXEml)

38. [Johansen AFB, Kassentoft CG, Knudsen M, Laursen MB, Madsen AH, Iversen LH, et al. Validation of computational determination of microsatellite status using whole exome sequencing data from colorectal cancer patients. BMC Cancer. 2019;19:971.](http://paperpile.com/b/N3ua4R/ePQWq)
