# Supplementary material for: Ultra-Deep Sequencing Reveals the Mutational Landscape of Classical Hodgkin Lymphoma
Source: Cancer Res Commun. 2023 Nov 15;3(11):2312–30. doi: 10.1158/2767-9764.CRC-23-0140 (PMC10648575; doi:10.1158/2767-9764.CRC-23-0140)
Supplement: Supplementary Figure 1 — Exome VAF and Depth of Coverage [file crc-23-0140-s02.docx]

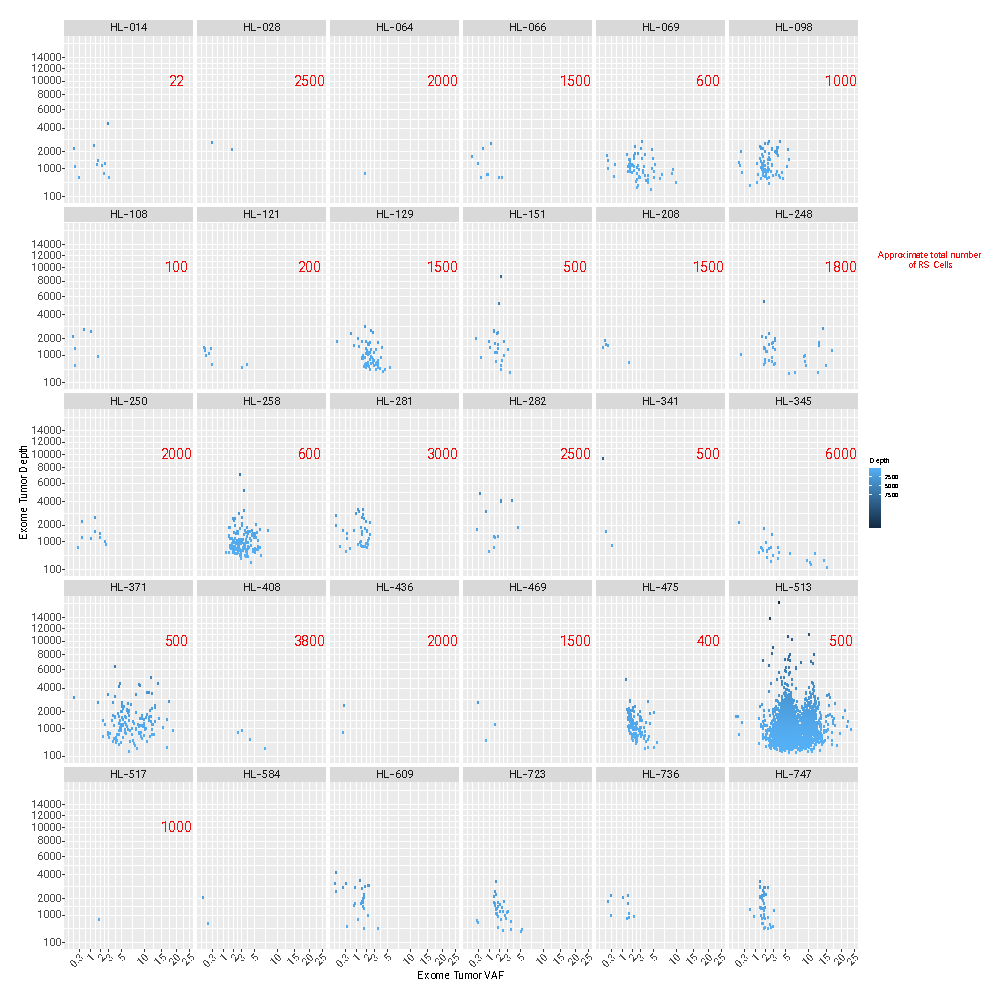


#### *Supplemental Figure 1. Exome VAF and Depth of Coverage*

A summary of all validated variants presented, plotted by sample. Each dot represents a variant. The variant allele frequency (VAF) and depth of coverage is shown. Dots are shaded based on exome depth. The numbers in red are the approximate number of Reed Sternberg cells (RS). We were unable to determine an approximate RS count for 5 samples. Patient HL-157 is not included because mutations from this patient were not included in any analyses.
