# Supplementary material for: Ultra-Deep Sequencing Reveals the Mutational Landscape of Classical Hodgkin Lymphoma
Source: Cancer Res Commun. 2023 Nov 15;3(11):2312–30. doi: 10.1158/2767-9764.CRC-23-0140 (PMC10648575; doi:10.1158/2767-9764.CRC-23-0140)
Supplement: Supplementary Figure 2 — Exome VAF and HaloPlex Tumor VAF [file crc-23-0140-s03.docx]

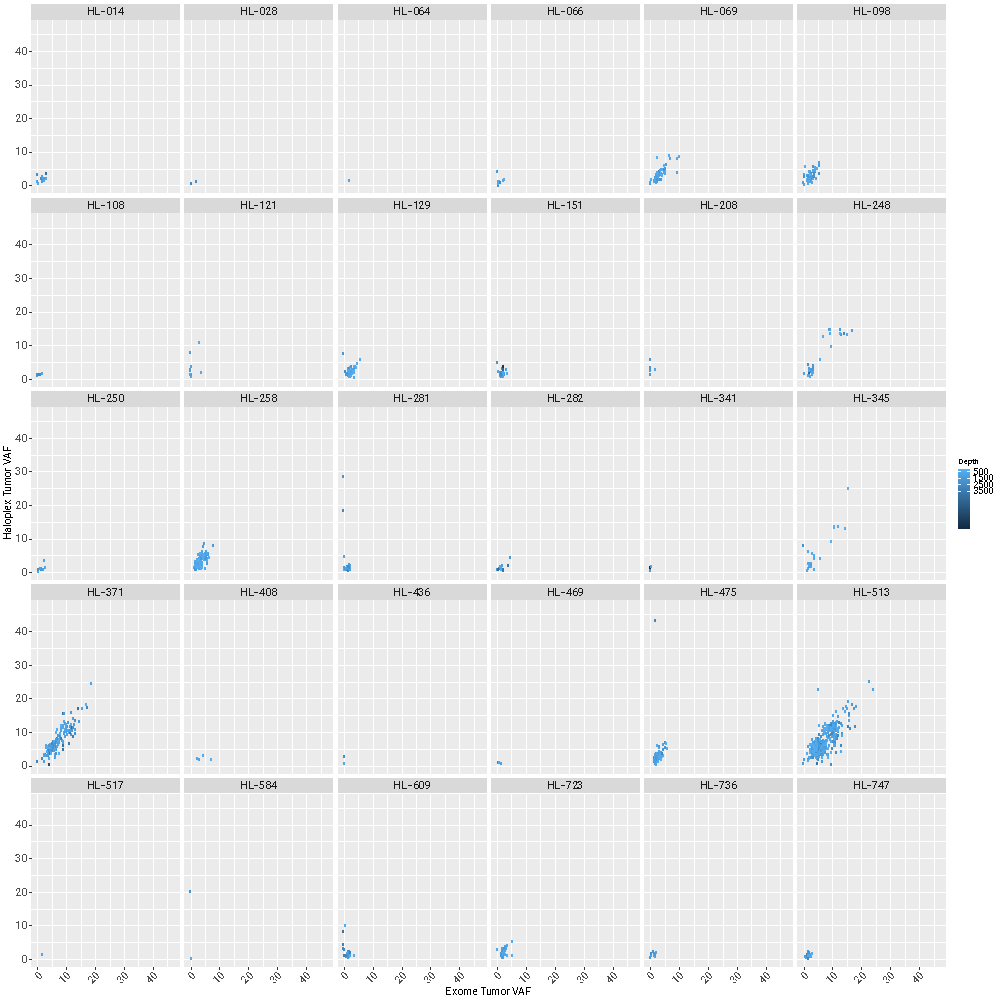


#### *Supplemental Figure 2. Exome VAF and HaloPlex Tumor VAF*

A summary of the HaloPlex and exome variant allele frequencies (VAF) for all validated variants, plotted by sample. Each dot represents a variant. Dots are shaded based on exome depth. Patient HL-157 is not included because mutations from this patient were not included in any downstream analyses
