# Supplementary material for: Ultra-Deep Sequencing Reveals the Mutational Landscape of Classical Hodgkin Lymphoma
Source: Cancer Res Commun. 2023 Nov 15;3(11):2312–30. doi: 10.1158/2767-9764.CRC-23-0140 (PMC10648575; doi:10.1158/2767-9764.CRC-23-0140)
Supplement: Supplementary Figure 3 — Relationship between Variant Validation Rate and The Number of HRS Cells/HPF [file crc-23-0140-s04.docx]

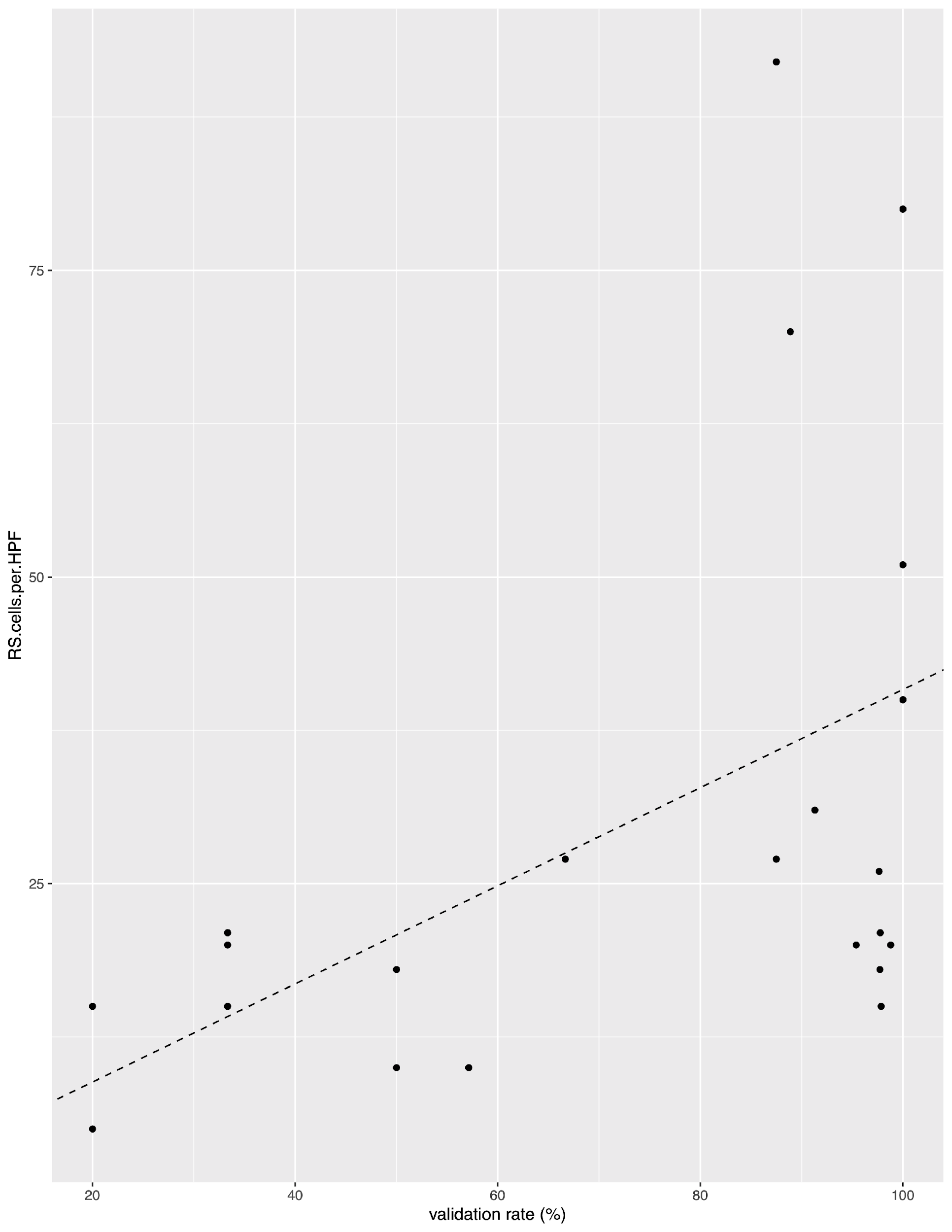


#### *Supplemental Figure 3. Relationship between Variant Validation Rate and The Number of HRS Cells/HPF*

We tested the relationship between the variant validation rate per sample (x-axis) and the number of HRS cells per HPF (high powered field) (y-axis). We observed a Pearson’s correlation of 0.50 between the sample validation rate and the number of HRS cells/HPF
