# Supplementary material for: Ultra-Deep Sequencing Reveals the Mutational Landscape of Classical Hodgkin Lymphoma
Source: Cancer Res Commun. 2023 Nov 15;3(11):2312–30. doi: 10.1158/2767-9764.CRC-23-0140 (PMC10648575; doi:10.1158/2767-9764.CRC-23-0140)
Supplement: Supplementary Figure 4 — Exome Tumor VAF Distribution Partitioned by Validation Status [file crc-23-0140-s05.docx]

*
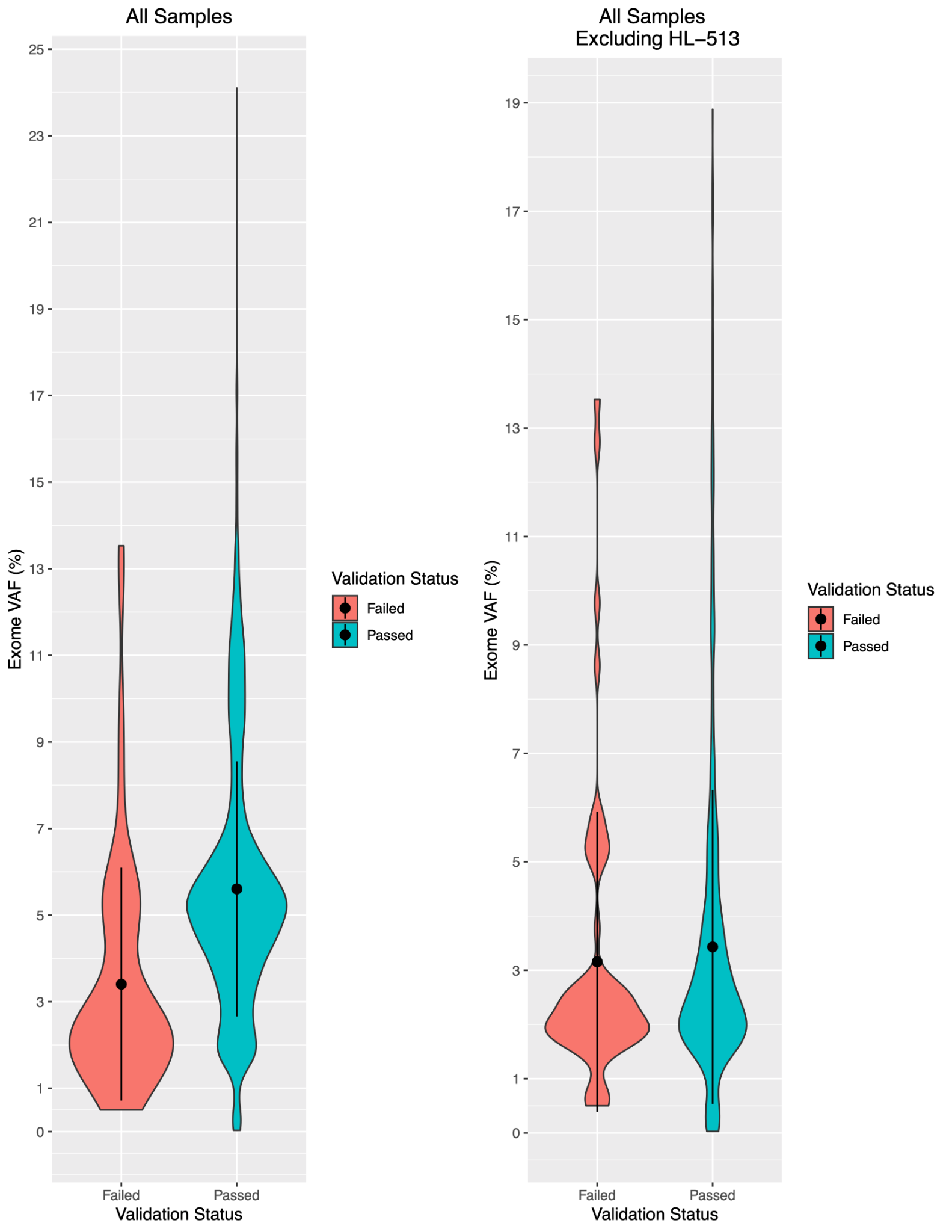
*

#### *Supplemental Figure 4. Exome Tumor VAF Distribution Partitioned by Validation Status*

A summary of the variant allele frequency (VAF) distribution of sites that passed HaloPlex validation and sites that failed HaloPlex validation in all samples (A) and in all samples excluding HL-513 (B). The red violins display the VAF distribution of sites that failed to validate, and the blue violins display the VAF distribution of sites that passed our validation criteria.
