# Supplementary material for: Ultra-Deep Sequencing Reveals the Mutational Landscape of Classical Hodgkin Lymphoma
Source: Cancer Res Commun. 2023 Nov 15;3(11):2312–30. doi: 10.1158/2767-9764.CRC-23-0140 (PMC10648575; doi:10.1158/2767-9764.CRC-23-0140)
Supplement: Supplementary Figure 5 — Exome Tumor VAF Distribution and Exome Tumor Coverage Partitioned by Validation Status [file crc-23-0140-s06.docx]

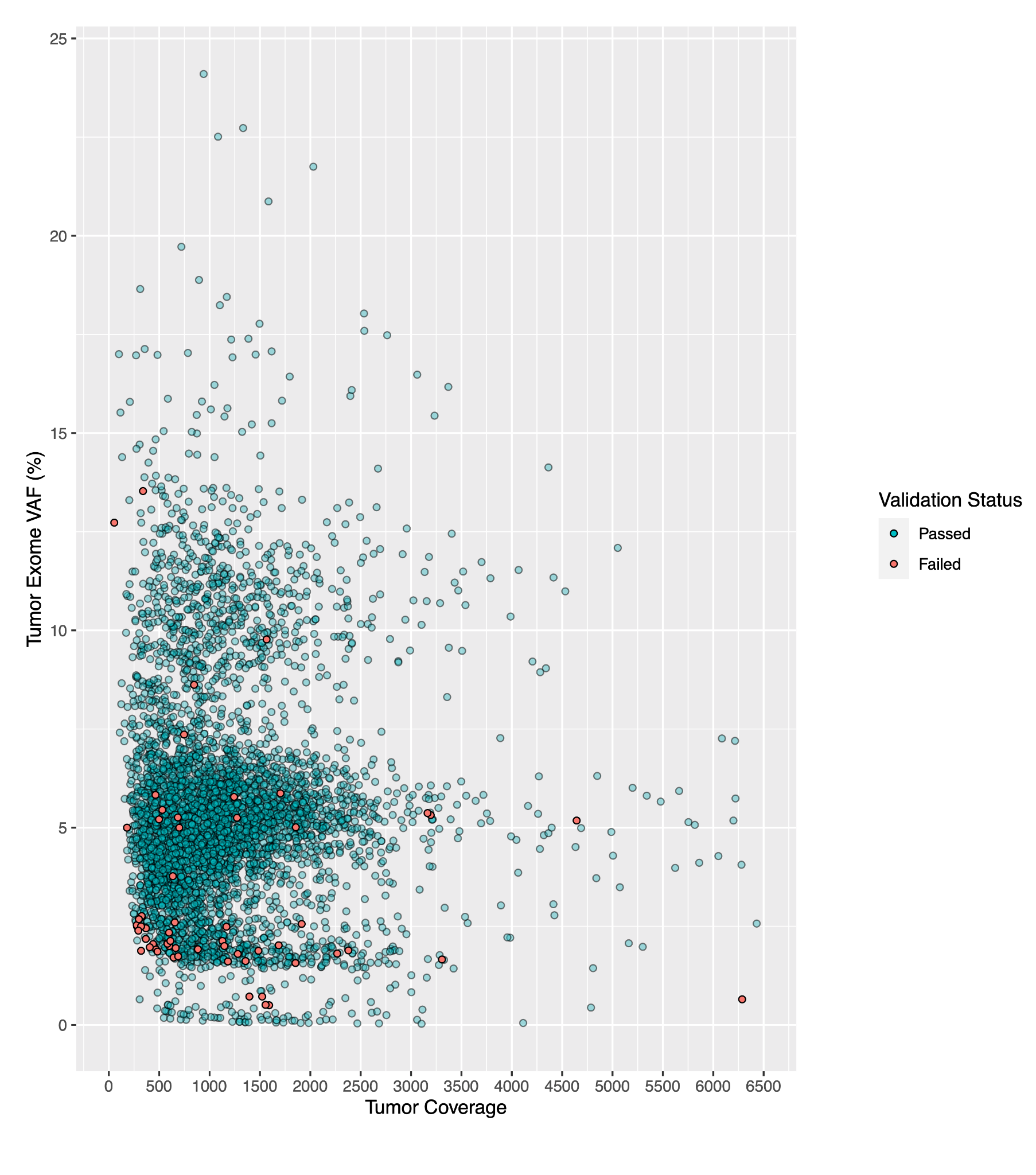


#### *Supplemental Figure 5. Exome Tumor VAF Distribution and Exome Tumor Coverage Partitioned by Validation Status*

A summary of variants discovered in the deep exome data. Each dot represents a variant. The variant allele frequency (VAF) and depth of coverage is shown. Dots are shaded based on validation status. Variants shaded in blue are variants that passed validation and variants shaded in red are those that did not pass validation. Note: 29 validated sites with outlier coverage values >6,500 reads (range: 6,586-17,498) are not shown.
