# Supplementary material for: Ultra-Deep Sequencing Reveals the Mutational Landscape of Classical Hodgkin Lymphoma
Source: Cancer Res Commun. 2023 Nov 15;3(11):2312–30. doi: 10.1158/2767-9764.CRC-23-0140 (PMC10648575; doi:10.1158/2767-9764.CRC-23-0140)
Supplement: Supplementary Figure 6 — Coverage Summary Across Sites that Passed and Failed Validation [file crc-23-0140-s07.docx]

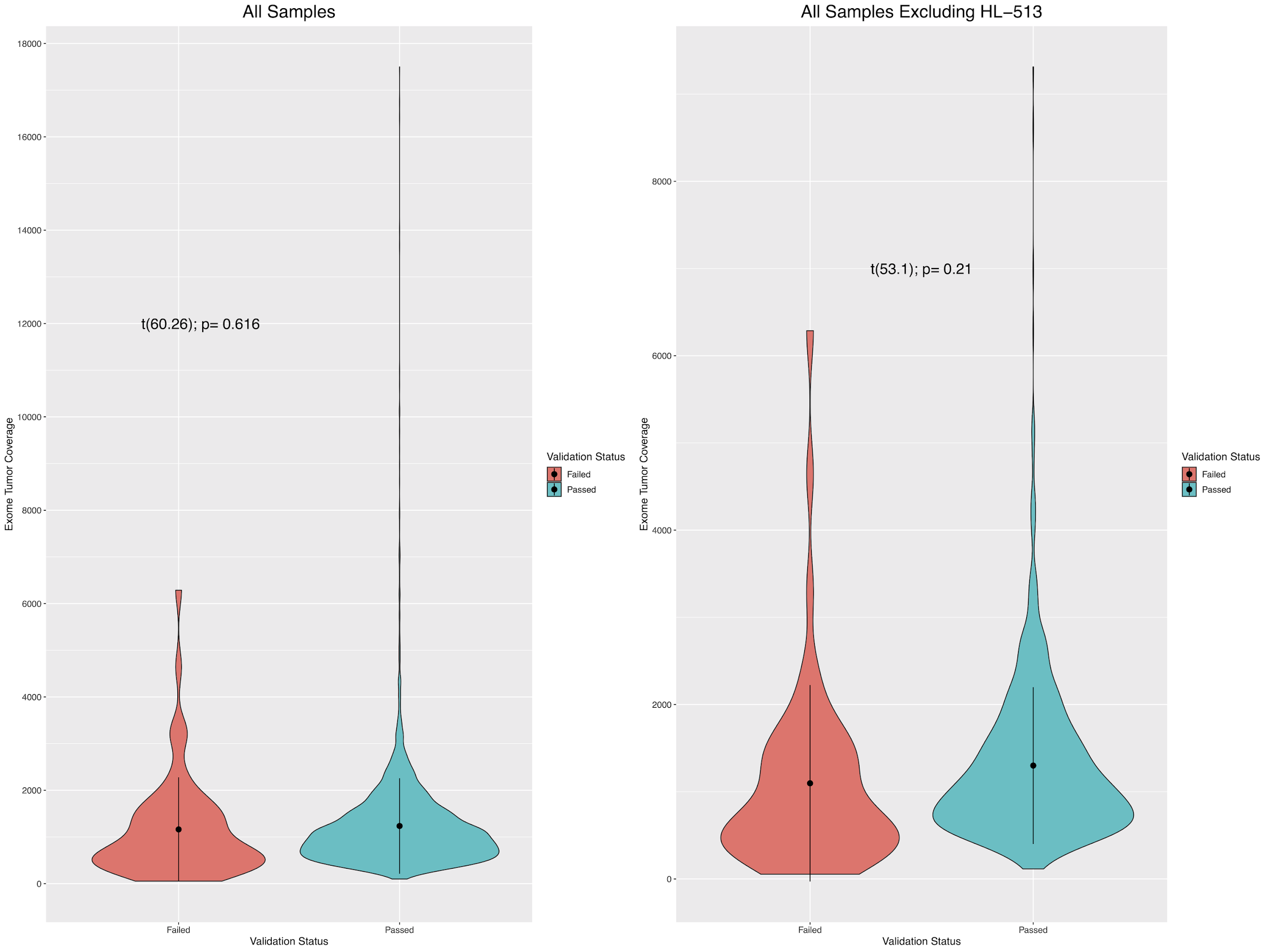


#### *Supplemental Figure 6.* Coverage Summary Across Sites that Passed and Failed Validation

A summary of the exome tumor coverage across sites that passed HaloPlex validation and sites that failed HaloPlex validation in all samples (A) and in all samples excluding HL-513 (B). The red violins display the coverage distribution of sites that failed to validate, and the blue violins display the coverage distribution of sites that passed our validation criteria.
