# Supplementary material for: Ultra-Deep Sequencing Reveals the Mutational Landscape of Classical Hodgkin Lymphoma
Source: Cancer Res Commun. 2023 Nov 15;3(11):2312–30. doi: 10.1158/2767-9764.CRC-23-0140 (PMC10648575; doi:10.1158/2767-9764.CRC-23-0140)
Supplement: Supplementary Figure 7 — figure 7Relationship between median Tumor VAF and Total Variant Count by Sample [file crc-23-0140-s08.docx]

*
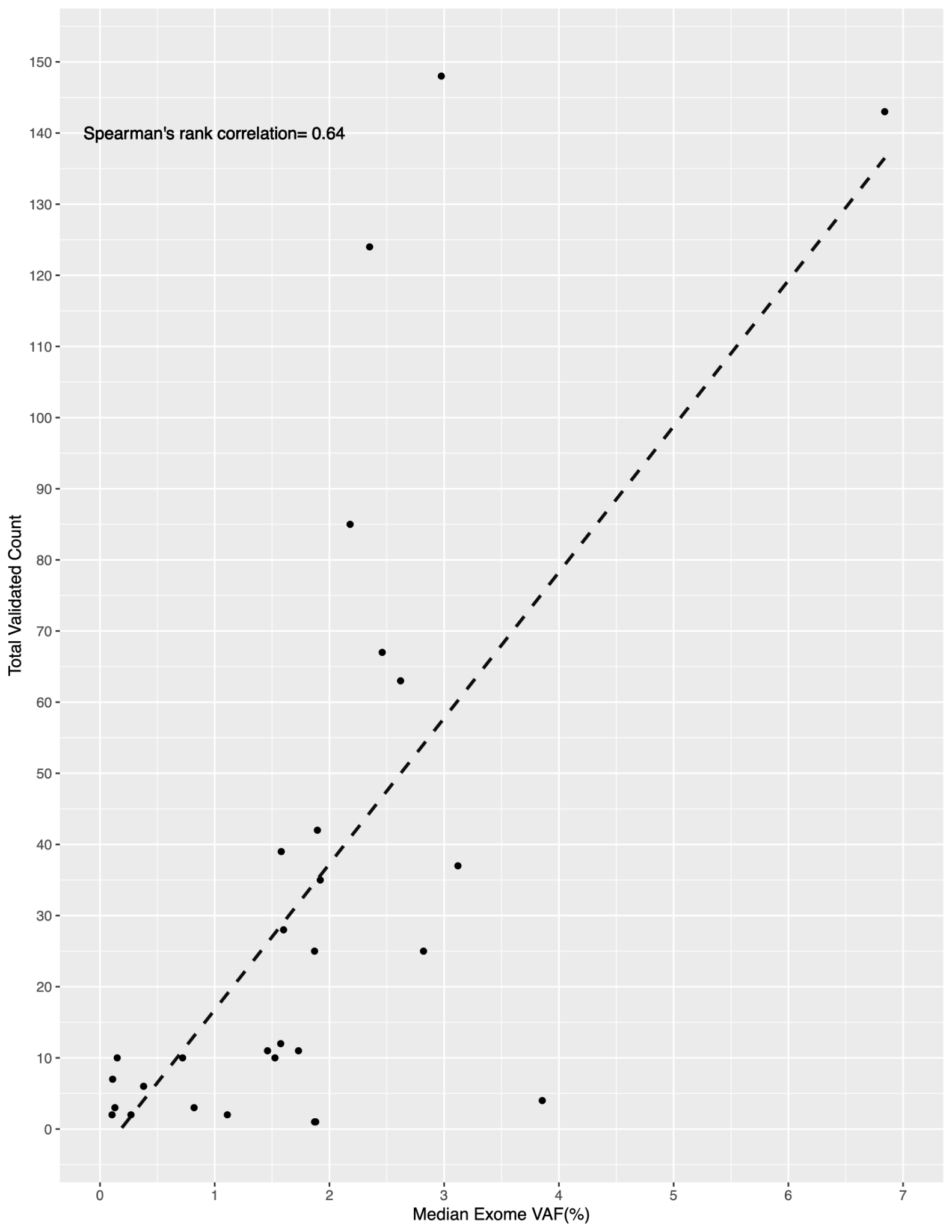
*

#### *Supplemental Figure 7. Relationship between median Tumor VAF and Total Variant Count by Sample*

We tested the relationship between the total number of validated variants per sample (y-axis) and the median tumor VAF (x-axis). We observed a correlation of 0.64 between total number of validated variants and median exome VAF.
