# Supplementary material for: Ultra-Deep Sequencing Reveals the Mutational Landscape of Classical Hodgkin Lymphoma
Source: Cancer Res Commun. 2023 Nov 15;3(11):2312–30. doi: 10.1158/2767-9764.CRC-23-0140 (PMC10648575; doi:10.1158/2767-9764.CRC-23-0140)
Supplement: Supplementary Figure 8 — Comparison of Genes Shown to be Recurrently Mutated in Studies of Adult cHL [file crc-23-0140-s09.docx]

*
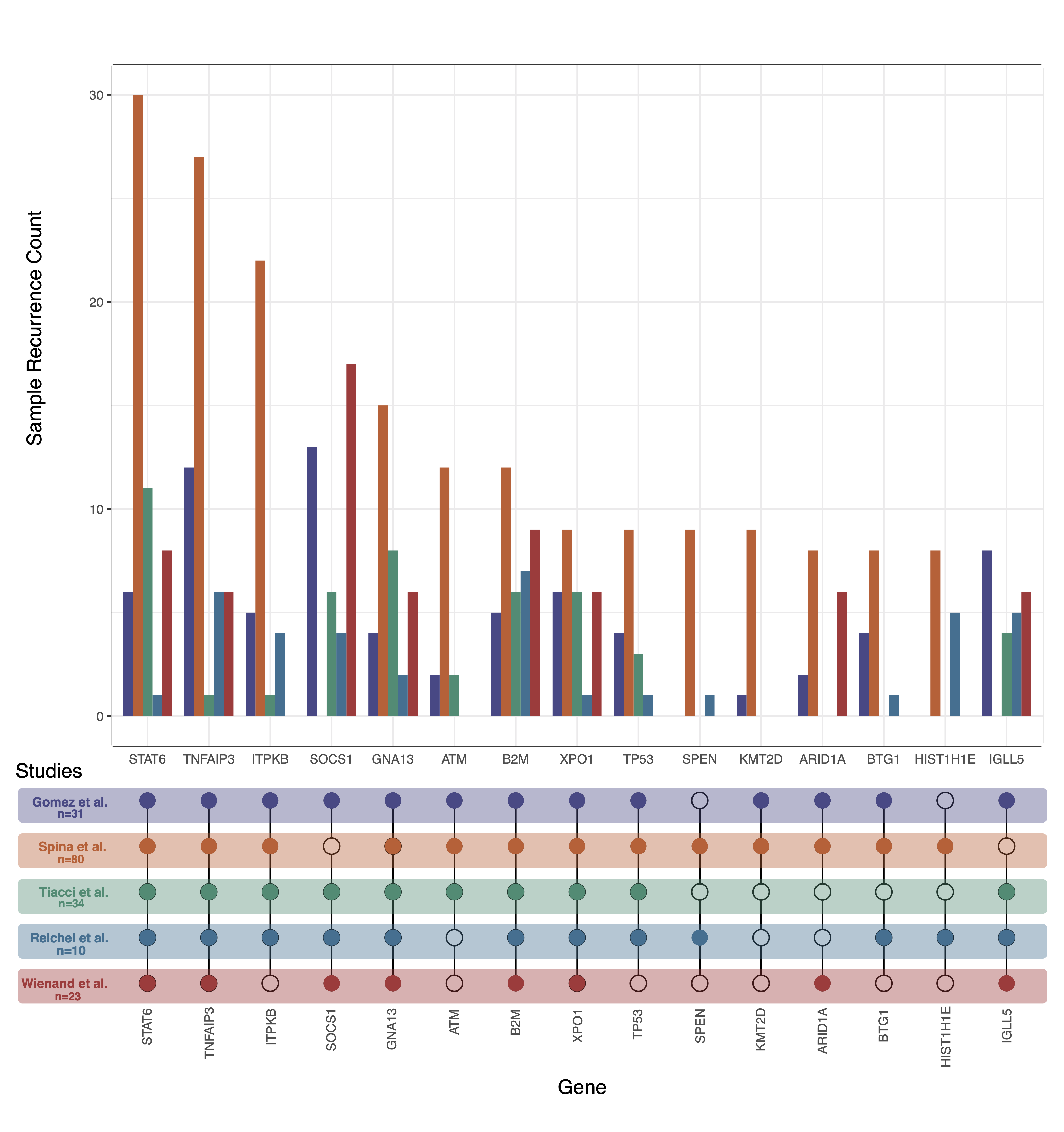
*

#### *Supplemental Figure 8. Comparison of Genes Shown to be Recurrently Mutated in Studies of Adult cHL*

The genes summarized here are the 15 most recurrently mutated genes across the 5 studies of the genomic landscape of adult cHL.(11,17–19) Each bar represents the recurrence of a particular gene, colored by study. The lower plot indicates whether each included study reported mutations in a selected gene.
