# Supplementary material for: Ultra-Deep Sequencing Reveals the Mutational Landscape of Classical Hodgkin Lymphoma
Source: Cancer Res Commun. 2023 Nov 15;3(11):2312–30. doi: 10.1158/2767-9764.CRC-23-0140 (PMC10648575; doi:10.1158/2767-9764.CRC-23-0140)
Supplement: Supplementary Figure 9 — Co-Occurrence and Mutual Exclusivity Among Significantly Mutated Genes [file crc-23-0140-s10.docx]

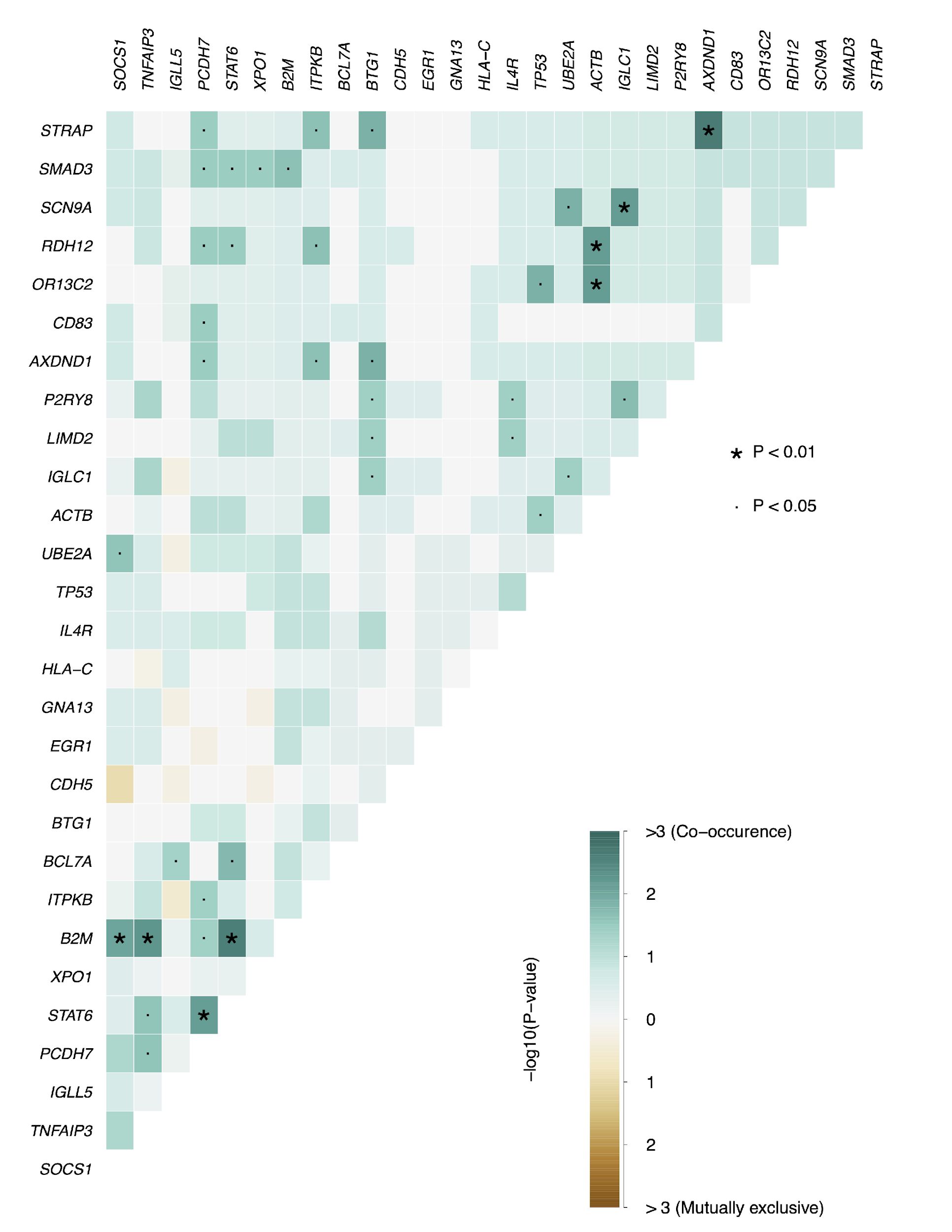


#### *Supplemental Figure 9.* Co-Occurrence and Mutual Exclusivity Among Significantly Mutated Genes

Co-Occurrence and Mutual Exclusivity Among Significantly Mutated Genes (inclusive of HL-513)

SMGs determined including HL-513 were examined for patterns of co-occurrence or mutual exclusivity.
