# Supplementary material for: Ultra-Deep Sequencing Reveals the Mutational Landscape of Classical Hodgkin Lymphoma
Source: Cancer Res Commun. 2023 Nov 15;3(11):2312–30. doi: 10.1158/2767-9764.CRC-23-0140 (PMC10648575; doi:10.1158/2767-9764.CRC-23-0140)
Supplement: Supplementary Figure 10 — Comparison of mutation burden and EBV status [file crc-23-0140-s11.docx]

#### *Supplemental Figure 10. Comparison of mutation burden and EBV status*

Mutation burden (mutation count) in samples that were found to be EBV positive (brown) or EBV negative (purple) using competitive alignment (Methods). The competitive alignment was largely concordant with EBV status determined using EBER ISH. Comparison of mutation burden between EBV positive and EBV negative patients using a t-test, excluding HL-513, indicates that there is not a significant difference in the mutation burden between the two groups (p=0.82).
