# Supplementary material for: Ultra-Deep Sequencing Reveals the Mutational Landscape of Classical Hodgkin Lymphoma
Source: Cancer Res Commun. 2023 Nov 15;3(11):2312–30. doi: 10.1158/2767-9764.CRC-23-0140 (PMC10648575; doi:10.1158/2767-9764.CRC-23-0140)
Supplement: Supplementary Figure 11 — Summary of Recurrently Mutated Genes for Relapsed Samples [file crc-23-0140-s12.docx]

**
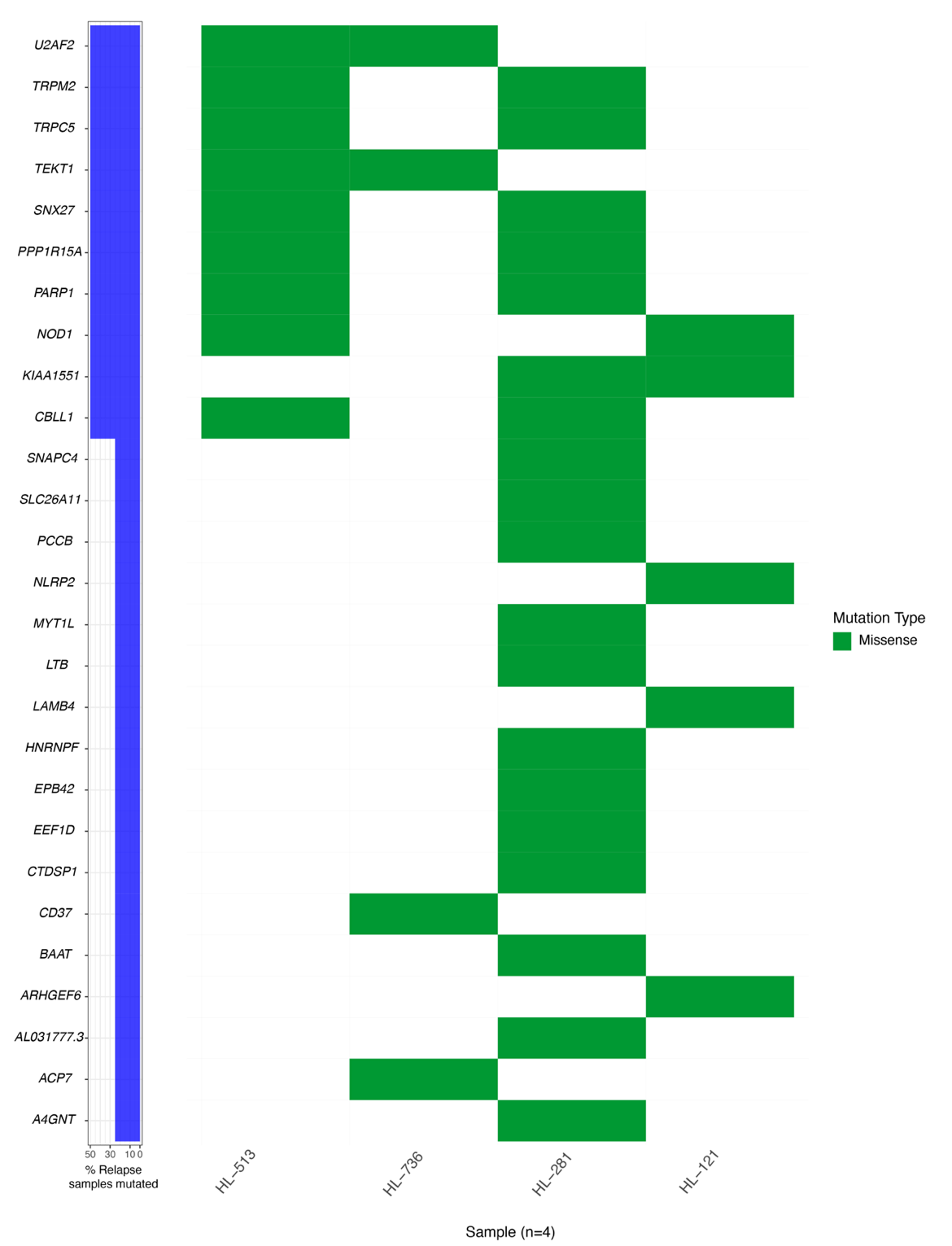
**

#### *Supplemental Figure 11.* Summary of Recurrently Mutated Genes for Relapsed Samples

#### The frequency and type of mutations found in the non-hypermutated relapse samples. Also included are genes mutated in the hypermutated sample and one additional relapse sample. Each column represents a relapse patient. The bar graph on the left summarizes the frequency of mutations for that gene across the relapse samples
