# Supplementary material for: Ultra-Deep Sequencing Reveals the Mutational Landscape of Classical Hodgkin Lymphoma
Source: Cancer Res Commun. 2023 Nov 15;3(11):2312–30. doi: 10.1158/2767-9764.CRC-23-0140 (PMC10648575; doi:10.1158/2767-9764.CRC-23-0140)
Supplement: Supplementary Figure 12 — Normalized Expression of EIF4A2 [file crc-23-0140-s13.docx]

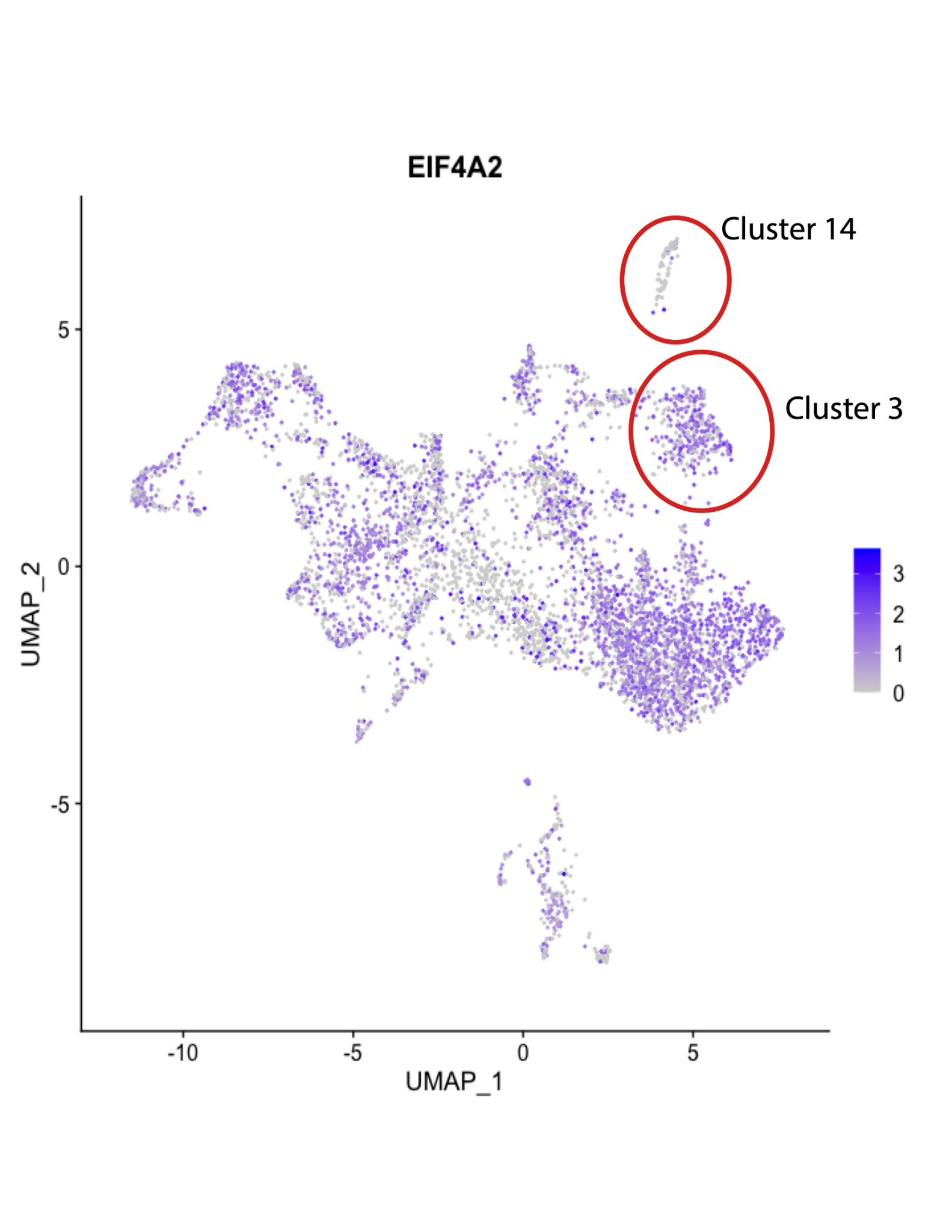


#### *Supplemental Figure 12. Normalized Expression of EIF4A2*

The normalized and scaled expression of EIF4A2 across all nuclei. The darker blue dots indicate nuclei with higher expression. The gray dots indicate nuclei where *EIF4A2* expression was not observed
