# Supplementary material for: Ultra-Deep Sequencing Reveals the Mutational Landscape of Classical Hodgkin Lymphoma
Source: Cancer Res Commun. 2023 Nov 15;3(11):2312–30. doi: 10.1158/2767-9764.CRC-23-0140 (PMC10648575; doi:10.1158/2767-9764.CRC-23-0140)
Supplement: Supplementary Figure 13 — HL-248 Reads Covering EIF4A2 in Cluster 14 [file crc-23-0140-s14.docx]

**
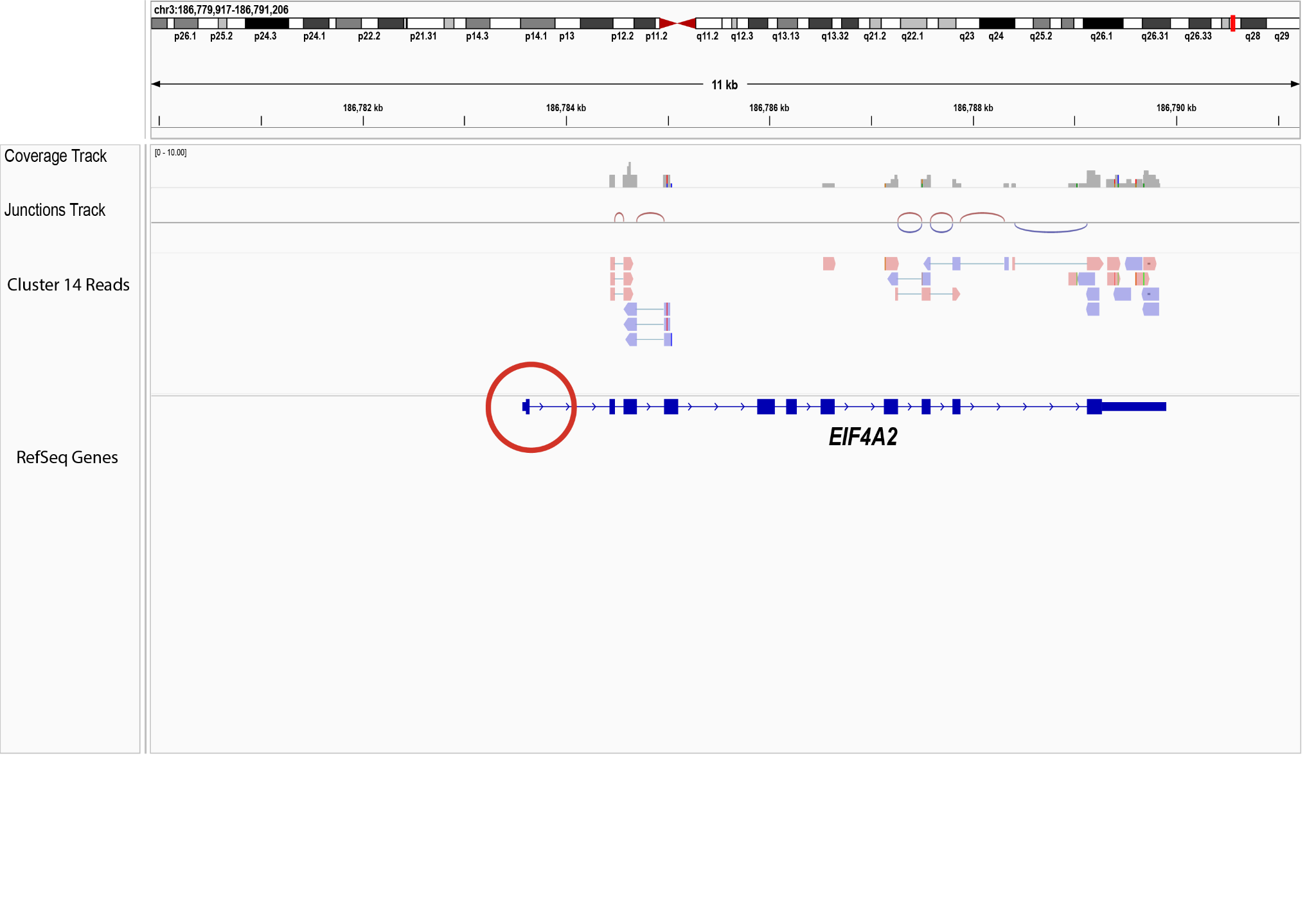
**

#### *Supplemental Figure 13.* HL-248 *Reads Covering EIF4A2 in Cluster 14*

#### An IGV image of the reads from HL-248 with barcodes annotated to cluster 14. The red circle indicates the location of exon one, which is the exon that contains the variant (ENST00000323963.9:c.24T>A; Y8*) identified in the deep exome of HL-248 and observed to be expressed in cluster 3.
