# Supplementary material for: Ultra-Deep Sequencing Reveals the Mutational Landscape of Classical Hodgkin Lymphoma
Source: Cancer Res Commun. 2023 Nov 15;3(11):2312–30. doi: 10.1158/2767-9764.CRC-23-0140 (PMC10648575; doi:10.1158/2767-9764.CRC-23-0140)
Supplement: Supplementary Figure 14 — Observed COSMIC v.3 mutation signatures [file crc-23-0140-s15.docx]

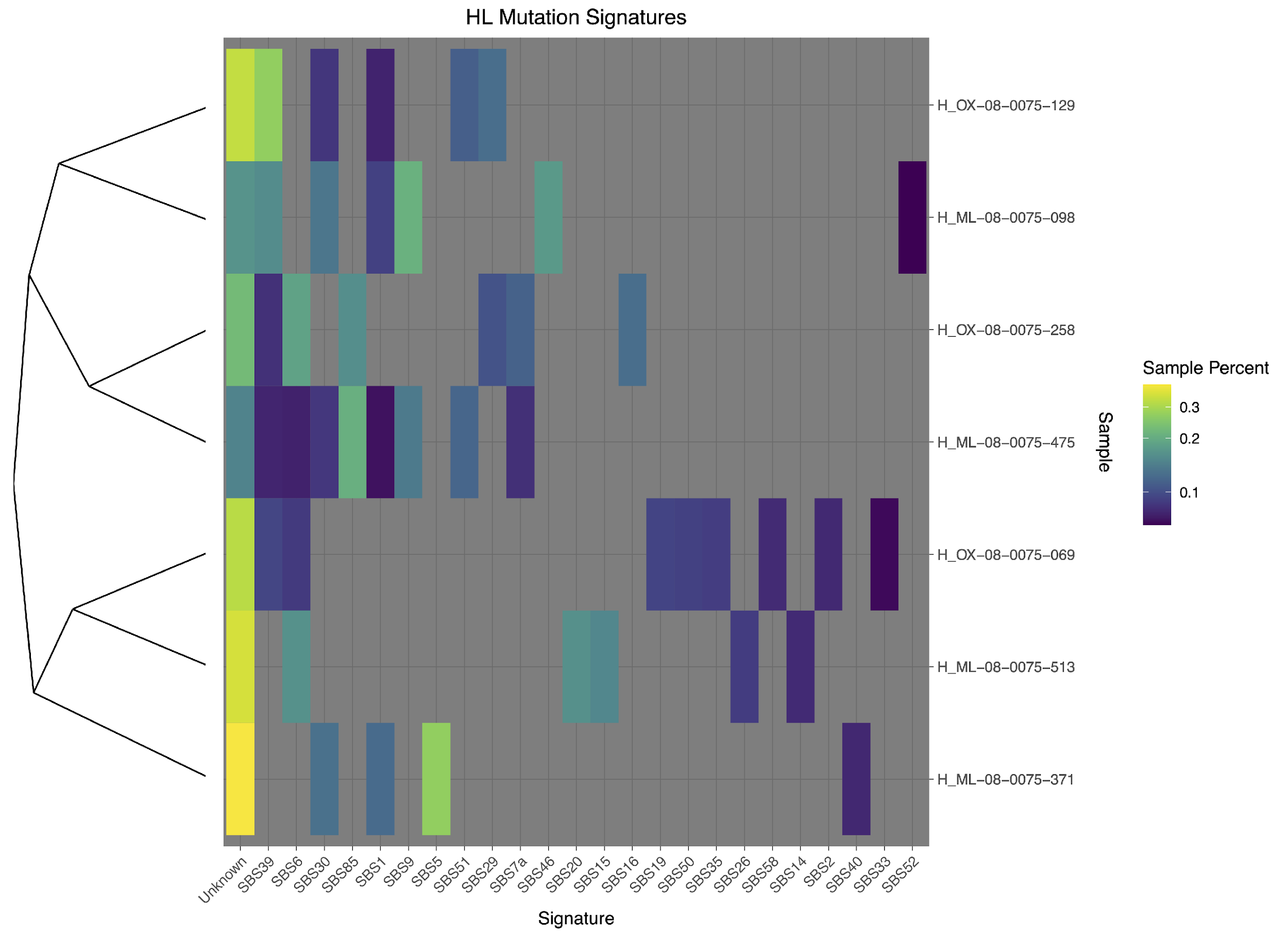


*Supplemental Figure 14. Observed COSMIC v.3 mutation signatures*

Patients included in this analysis had at least 50 somatic mutations. Shading represents the observed proportion of a particular signature out of all signatures observed in that sample. Dendrogram represents sample relatedness based on similarity of mutation signatures.
