# Supplementary material for: Ultra-Deep Sequencing Reveals the Mutational Landscape of Classical Hodgkin Lymphoma
Source: Cancer Res Commun. 2023 Nov 15;3(11):2312–30. doi: 10.1158/2767-9764.CRC-23-0140 (PMC10648575; doi:10.1158/2767-9764.CRC-23-0140)
Supplement: Supplementary Figure 15 — VAF Distribution of JAK/STAT and Hippo/TAZ/YAP regulation associated variants [file crc-23-0140-s16.docx]

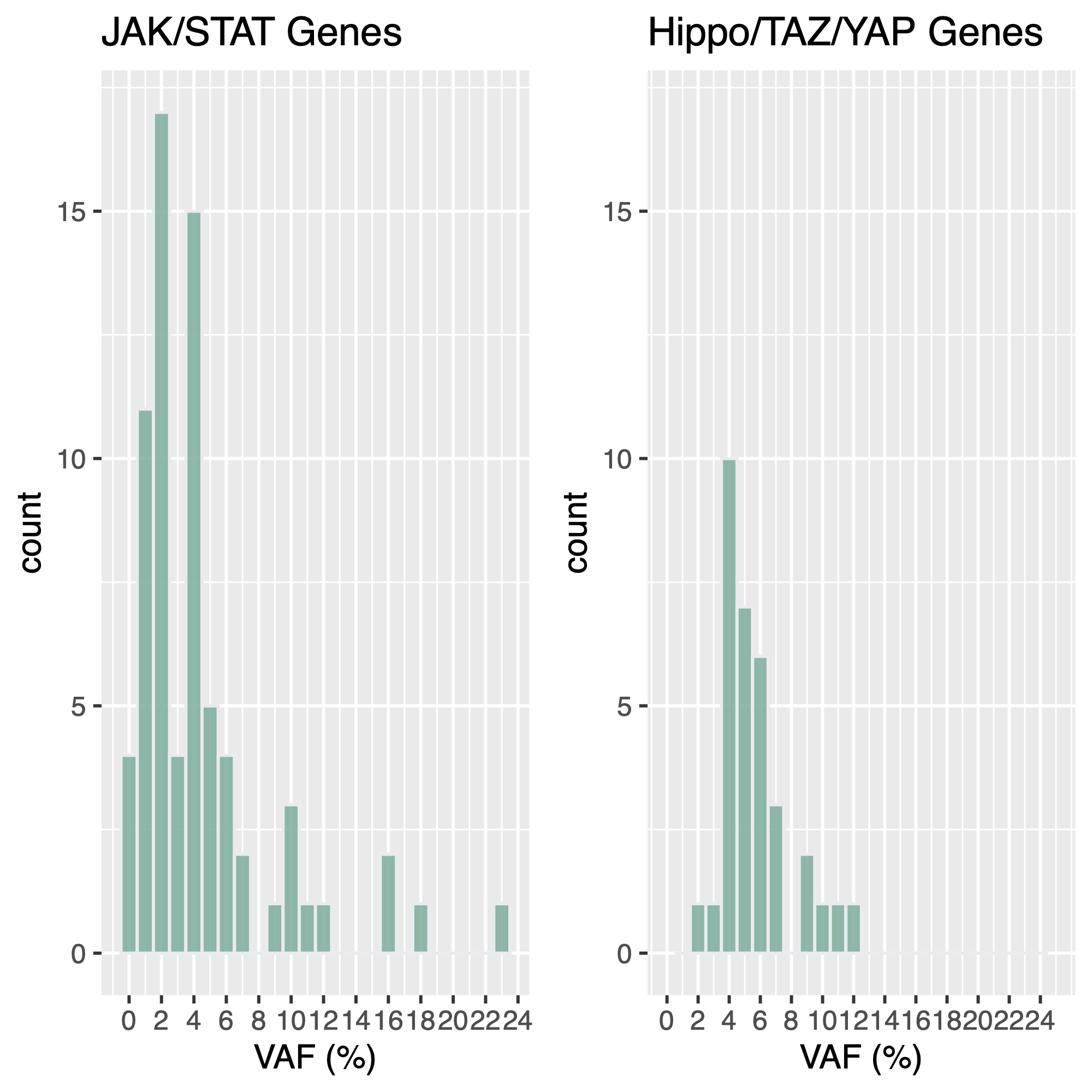


#### *Supplemental Figure 15.* VAF Distribution of JAK/STAT and Hippo/TAZ/YAP regulation associated variants

#### Distribution of variants VAFs associated with the JAK/STAT and Hippo/TAZ/YAP regulating pathways. The JAK/STAT pathway (mean = 4.4%; defined by KEGG) and the Hippo/TAZ/YAP regulatory pathway used here (mean = 4.8%; defined by WikiPathways) are similar, despite there are overall more variants attributed to the JAK/STAT pathway
