# Supplementary material for: Ultra-Deep Sequencing Reveals the Mutational Landscape of Classical Hodgkin Lymphoma
Source: Cancer Res Commun. 2023 Nov 15;3(11):2312–30. doi: 10.1158/2767-9764.CRC-23-0140 (PMC10648575; doi:10.1158/2767-9764.CRC-23-0140)
Supplement: Supplementary Figure 16 — Diagram of observed mutated components of the MAPK signaling cascade [file crc-23-0140-s17.docx]

*
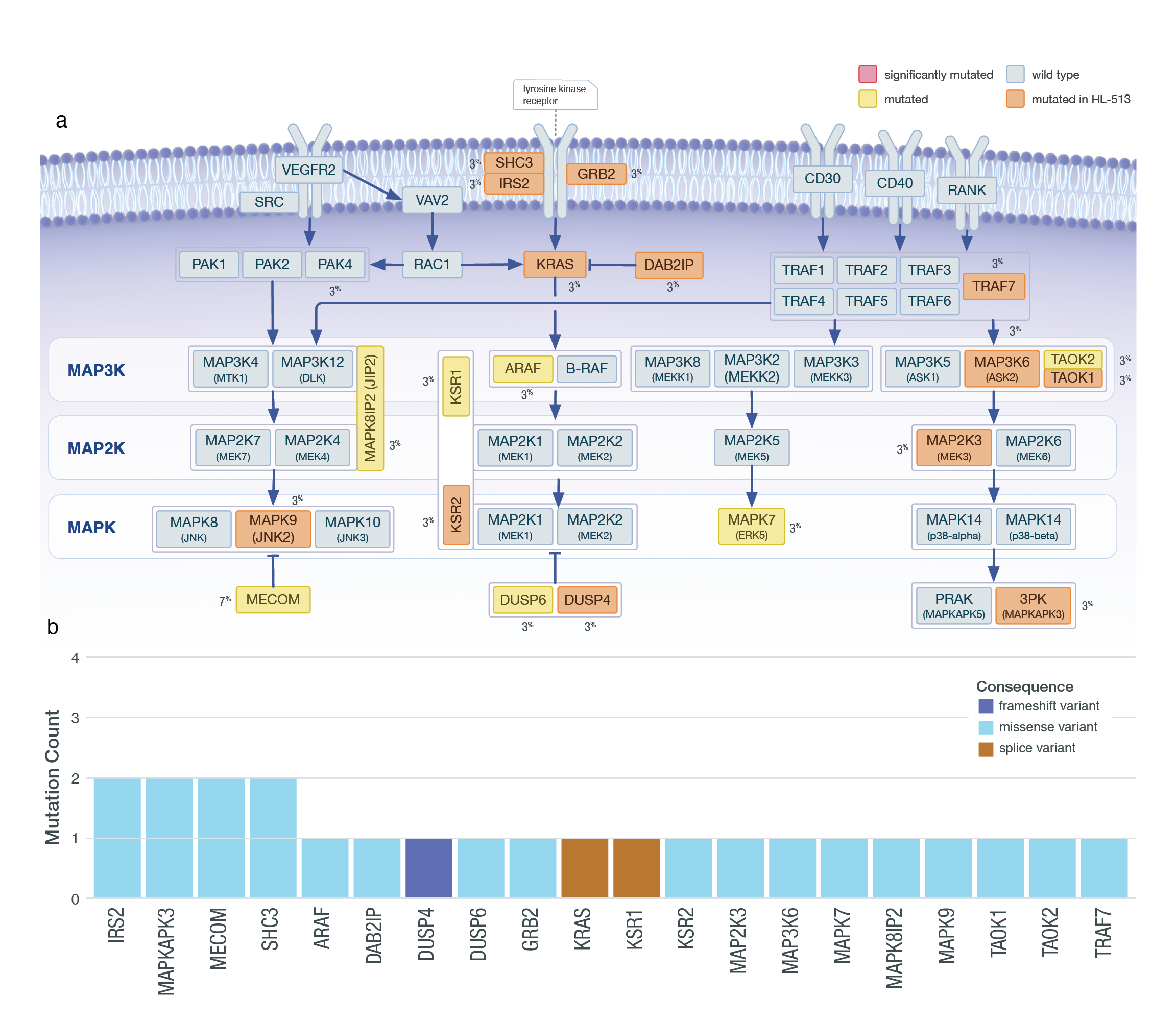
*

#### *Supplemental Figure 16. Diagram of observed mutated components of the MAPK signaling cascade*

a) Genes mutated only in the hypermutated sample are shown in orange; genes mutated in at least one non-hypermutated sample are shown in yellow; genes that were identified as SMGs are shown in red. The frequency of the gene mutated across the cohort is shown as a percent. b) The total number and type of mutations observed are shown in the inset bar chart.
